# Potential differences between the political attitudes of people with same-sex parents and people with different-sex parents: An exploratory assessment of first-year college students

**DOI:** 10.1371/journal.pone.0246929

**Published:** 2021-02-25

**Authors:** Andrew R. Flores, Maisy Morrison

**Affiliations:** 1 Department of Government, School of Public Affairs, American University, Washington, Columbia, United States of America; 2 Department of Public Policy and Political Science, Mills College, Oakland, California, United States of America; University of California-Irvine, UNITED STATES

## Abstract

Children were often near the center of public debates about legal marriage recognition for same-sex couples. *Obergefell v*. *Hodges* (2015), the case that resulted in legal same-sex marriage recognition, stressed the importance of these children as one of many factors compelling the opinion. Estimates indicated same-sex couples were raising 200,000 children in the United States. Children raised by same-sex couples may be politically socialized in distinct ways compared to children of different-sex couples because lesbians, gay men, and bisexuals tend to hold distinct and progressive political viewpoints on a wide variety of issues. What are the political attitudes of people with same-sex parents? In this exploratory study, we analyze a large, representative survey of first-year college students across the United States; we find few differences between people with same-sex and different-sex parents, and some of those differences may be attributable to households and respondent characteristics. When on the rare occasion a difference exists, we find that people with same-sex female parents are more progressive, but people with same-sex male parents are more conservative. Gender differences also emerged, with some distinctive patterns between males with same-sex parents and females with same-sex parents.

## Introduction

On June 26, 2015, Justice Anthony Kennedy rendered the majority opinion in *Obergefell v*. *Hodges* and in effect legally recognized marriages for same-sex (SS) couples across the United States. In his opinion and in oral arguments, Justice Kennedy seemed preoccupied by the sheer number of children being raised in SS households. It would only later come out how much the children mattered to him: “it just seemed to me…wrong under the constitution to say that over 100,000 adopted children of gay parents couldn’t have their parents married” [1].

About 200,000 children are being raised in SS couple households, and three million lesbian, gay, bisexual, and/or transgender (LGBT) adults in the US have had a child [2]. Over half of them are biological children [2]. LGBT parents tend to be people of color and have lower median household incomes [2]. There remain research gaps in the systematic study of people with SS parents [3], and most of this research is in the domain of developmental and educational psychology. In that context, most studies show that children and adolescents of SS couples are not at any disadvantage when compared to children and adolescents of different-sex (DS) couples [4–7]. We explore the political attitudes of people with SS parents and compare them to those of people with DS parents. We find that the political attitudes of people with SS parents do not differ much from people with DS parents both before and after considering household and demographic characteristics. When differences exist, people with SS male parents tend to be more conservative and people with SS female parents tend to be more progressive than their counterparts.

Political socialization is the process through which young people form their political attitudes and behaviors [8]. Parents are a large contributor to political socialization. The two major schools of thought in American political behavior identify the family as a primary source of a person’s political attitudes and behaviors [9,10]. There is a positive correlation between the attitudes of young adults and their parents, but they are not consistently strong [11]. Parental socialization is stronger if families are highly politicized and parents consistently share their political viewpoints [12]. Parents provide their children with prior beliefs about politics, and beliefs are updated by other experiences [13].

There is ample reason to believe that people with SS parents are socialized in an environment that would result in distinct political attitudes. LGB people are more politically engaged and socially progressive [14–19]. LGBT people are more likely than non-LGBT people to engage in political discussion, write government officials, attend protests and rallies, and give money to political campaigns [15]. LGBT people are more progressive on issues beyond LGBT rights including, abortion, gun control, immigration policy, environmental protection, criminal justice reform, and race-relations in the US [15]. About 75% of LGBT people identify with or lean toward the Democratic Party compared to 45.2% of non-LGBT people [15]. Such political distinctiveness may pass to children of SS couples, especially because research shows that SS parents spend on average more time with their children than DS parents [20].

Gay and lesbian parents also engage in conversations with their children about homophobia and legal inequities [21–25]. Conversations can build a family identity [21,22], foster resilience [24], and may be motivated by parents’ concerns over the impact sexual stigma may have on their children [23]. One qualitative study in Florida finds that gay and lesbian parents who have these conversations do so to prepare their children for anti-gay stigma and discrimination, to foster pride, and to encourage political activism [25]. These conversations better enable children to cope with adversity to homophobia [23] and stigma as it relates to their family structure [21]. Gay and lesbian parents tend to have these conversations when they believe their children are at an appropriate age when it seems necessary that they may begin to become aware of social stigmas around homosexuality [25]. These studies suggest that the sociopolitical socialization of people with SS parents is distinct from people with DS parents due to social stigmas and discrimination LGB people encounter.

Major political events that occur during adolescence can also have lasting effects on youth partisanship [26]. Recently in LGBT rights, major political events occurred that likely reinforce the importance of the rights of SS couple households. The data for this study come from the same year as the *Obergefell* decision, which followed the rulings of several federal district and circuit courts legally recognizing marriages for same-sex couples. These major legal and political events improved the wellbeing of LGB households [27], and disparities in measures of wellbeing between LGBT and non-LGBT people were reduced immediately following the *Obergefell* decision [28]. Events such as these can have long-lasting effects on youth political orientations [26], likely increasing the relevance of progressive politics and LGBT rights for the wellbeing of people with SS parents and their parents. Indeed, it was after Florida changed its adoption law in 2010 that gay and lesbian parents took the opportunity to discuss legal inequities with their children [25].

Previous studies also support the expectation for people with SS parents to have distinctive experiences affecting their political attitudes. Qualitative studies suggest that adults who have LGB parents tend to report that they feel more open-minded, more impacted by laws regulating family, and more affected by the prejudices their parents face [29–31]. For example, some people with SS parents questioned whether they would themselves marry when legal marriage was denied to their parents [30]. These patterns are similar for children in multiracial households who have a different set of political attitudes [32].

For these reasons, we expect:

H1: People with SS parents will be more progressive than people with DS parents.

There are gender differences in the way males and females are politically socialized [33], which influences their political attitudes [33] and behaviors [34]. In the general population, households with girls tend to more strongly instill traditional gender role beliefs and be Republican leaning [35]. Politics tends to be viewed and internalized by women and girls as a male-dominated space particularly as young girls grow older [36], and they develop disinterest toward politics [37], which may lower their ambition to run for public office [38]. Though contextual factors can reduce some of these differences such as having a female social studies teacher [39], the differences in the political socialization of boys and girls continues to result in gaps in their political attitudes and behaviors [33].

There are additional gender differences of children of SS parents in their psychological adjustment [e.g, 40]. For instance, adolescents raised with lesbian parents occasionally have gender differences in measures of their psychological adjustment [40]. We can only conjecture with the existing literature about the possibility for having SS parents to result in differences between men and women, though we consider gender to be an important consideration for analysis. Given the gender differences in political socialization and in psychological adjustment, we consider the following:

RQ1: Does respondent gender moderate the effect of having SS parents?

In addition, male and female sexual minorities internalize social stigmas and are sensitive to sexual stigma to different degrees [41]. Thus, SS male households may be different from SS female households in how they discuss homophobia and discrimination [41]. As detailed in the next section, we further subdivide our analyses between SS female parents and SS male parents. This latter decision is motivated by prior research [4] and following recommend practices due to the differences between SS male and female couples in the family formation process [42,43]. While this decision is motivated by analytic best practices, the gender of the SS couple is another important dimension to our results.

## Materials and methods

The data for this exploratory study come from the 2015 administration of The Freshman Survey conducted by the Higher Education Research Institute (HERI) at the University of California at Los Angeles. Every year, HERI conducts surveys of first-year college students among participating American colleges and universities. The multistage, complex survey collects demographic and financial data about first-year college students and their parents throughout the United States. All institutions of higher learning that have a new first-year cohort in a degree granting program at the baccalaureate-level or higher are invited to participate, and all first-year, full-time students at these institutions are recruited to participate. HERI charges fees to these institutions to administer the survey, and member institutions have access to data and reports specific to their institution. In 2015, 199 colleges and universities participated with at least 65% of the first-year cohort completing the survey. The survey was administered between March and October and interviewed 141,189 first-time, full-time first year students [44]. The data are weighted by stratifying by institution type, then by gender of the first-year cohort, and then the demographics are adjusted for the demographics within each institution strata. Access to the data is restricted by HERI, but investigators can apply for data access by articulating a research design, hypotheses, and planned use of the data.

### Measures

#### Same-sex and different-sex households

Beginning in 2015, the HERI modified its questionnaire from “Father/Mother” to “Parent 1/Parent 2,” and added the item: “Please mark the sex of your parent(s) or guardian(s).” As a result of this change, the 2015 HERI is one of the largest surveys of people who self-report SS parentage in the United States. There were 602 respondents reported having SS female parents, 176 respondents reported having SS male parents, and 134,142 respondents reported have DS parents. The item is imperfect since it is self-reported, and self-reports like these should be handled with caution and be used to operationalize concepts clearly [e.g., 45,46]. The present question does not determine whether one of the parents is biological or if the respondent was fostered or adopted. Despite these limitations, the present measurement approach is likely the best for self-reported surveys and reflects practices common to household surveys, where one individual reports on characteristics of others in the household.

#### Dependent variables

The HERI Freshman survey included a battery of questions on politics. This included ideological self-placement and nine policy-related questions including concerns over racial discrimination, abortion, gender workplace equality and women’s equal pay, free speech on college campuses, affirmative action policies in college admissions, the United States intervening in foreign conflicts, marijuana legalization, and marriage equality. Ideology was measured on a 5-point scale from “Far Right” to “Far Left.” The policy-related questions were measured on a 4-point scale from “Strongly Disagree” to “Strongly Agree.” Most studies indicated that LGBT people are far more politically progressive than non-LGBT people across a wide variety of issues, so we explored all these items. The factor structure of these measures, which was initially assessed to reduce multiple comparisons, did not support combining these measures into one or more scales. Instead, all these variables were rescaled to range from zero to one with higher values indicating more politically progressive viewpoints. Question wordings and associated variable names are in S1 Appendix.

#### Respondent demographic characteristics

We considered the following demographic characteristics of the individual respondents: gender (female = 1), sexual orientation (heterosexual = 1), first generation status (first generation = 1), household income (14 categories ranging from less than $10,000 to $250,000 or more), age (10 categories ranging from 16 years old or younger to 55 years old or older), race or ethnicity (7 categories including White, Black, Hispanic, Asian, American Indian, Biracial, or Other), and religious affiliation (8 categories as listed in S1 Appendix). About 55% were female, 93% were heterosexual, 17% were first generation, the median age was 18 years old and 68% were 18 years old, the median household income was between $75,000-$99,999, 58% were white, 13% were biracial, 10% were Asian, 10% were Hispanic, and the modal religious category was “none” and 20% had no religious affiliation.

#### Parent demographic characteristics

The HERI obtained information about each respondent’s parent. In addition to each parent’s sex, we considered the following: parents’ educational attainment, the relationship status of the parents (e.g., living together, living apart, or if one parent is or both are deceased), employment status (3 categories including employed, unemployed, or retired), and religious affiliation. About 9% of the respondents had parents where both had a graduate degree, 17% had parents where both had a college degree, 25% of the respondent’s parents were divorced or living apart, 71% of the respondent’s parents were living with each other, 68% had both parents employed and 97% had at least one parent employed, and the modal religious affiliation of the parents was Roman Catholic. We note that 80% of the respondents with SS male parents had both parents living together, but 31% of the respondents with SS female parents had both parents living together.

### Analysis

We followed standard procedures when analyzing large-*N* survey data of analyses comparing people with SS parents to people with DS parents. We first restricted our analyses to only respondents who reported having two parents because there is ample evidence that two-parent households differ from single-parent households [see e.g., 4]. We also stratified the analysis by household types (female and male SS parent versus DS parent households), because there were meaningful differences, on average, between the pathways that female SS parents and male SS parents come to be parents [42,43]; this was also consistent with previous approaches [4]. For appropriate standard error estimation, survey strata and primary sampling units were used in addition to two estimates of survey weights. For overall comparisons, the probability weights provided in the HERI data were used, which should provide representative estimates.

In addition, we considered the background differences between people in SS parent and DS parent households. Those who identify as LGB tended to have background characteristics that might make them seem even more progressive when those characteristics were not considered [14]. For example, identifying as LGB was more likely for individuals who have politically progressive parents; thus, a more appropriate comparison would examine straight people who have similarly progressive parents [14]. Similar concerns could be raised for people with SS parents because there might be background characteristics that are not similar between SS and DS parent households. Consistent with previous studies [4], a statistical matching procedure was performed. We used propensity score weights from a covariate balanced propensity score (CBPS) model to adjust the sample of people with DS parents to match the characteristics of the people with SS parents [47]. CBPS was a robust estimation approach to bring balance between two groups; the method provided a flexible estimation that can handle high-dimensional data without overfitting [see 47, p. 246]. The propensity score model included the respondent’s demographics and characteristics of parents such as relationship status, employment, educational attainment, household income, and religion. The CBPS model was fit in R v. 3. 5. 1 relying on the CBPS package [48]. The results of the CBPS estimation were provided in S2 Appendix, and Figure S2.1 in S2 Appendix shows substantial imbalance between SS and DS parent households before weighting and significant reductions in imbalance after weighting. For analysis, we also used the inverse propensity score multiplied by the probability weights, following recommended best practices [49]. When we described results, analyses based on probability weights calculated by the HERI were referred to as weighted and unmatched results, and analyses based on the addition of the CBPS weights were referred to as the weighted and matched results. Aside from the matching process, all remaining analyses were performed in Stata SE v. 14.1

We provide demographic summaries between people in female and male SS parent households as compared to people in DS parent households. To explore political attitudes, mean scores on the dependent variables are provided relying on both the probability weights and the final weights to compare people with SS and DS parents. The results reported are largely consistent when controlling for respondent demographics as in S3 Appendix, and whether or not weights are used as in S5 Appendix. To examine gender differences, we followed recommended practices to treat propensity score weighting as a data preprocessing step, and regression was then used to adjust for respondent characteristics interacting respondent gender and household type [50,51]. Model predictions of the dependent variables are then reported by sex of the respondent for people with SS and DS parents, controlling for age, race or ethnicity, LGBT identification, first generation status, and household income; the full model results are in S3 Appendix. Ideology is also controlled for in policy opinions. These results are not sensitive to the use of particular weights or whether regression adjustment is used.

## Results

### Demographic characteristics

Table 1 provides the demographic characteristics by household type. Prior to matching, people with SS female parents are less heterosexual, more female, more first generation, lower income, slightly older, less likely to racially self-identify as white and more likely to racially self-identify as black compared to people with DS parents. People with SS male parents are less heterosexual, less female, slightly older, and more likely to racially identify as Asian compared to people with DS parents. There are also differences between people in SS female and SS male households. People with SS female parents are more heterosexual, more female, more first generation, lower income, less likely to be racially self-identify as white or Asian and more likely to racially self-identify as black compared to people with SS male parents. All the demographic differences between people with SS parents and DS parents are statistically insignificant after matching as documented in S2 Appendix. Given the greater propensity for people who self-report SS female parents to racially self-identify as black, the HERI administrators suspected that extended family members may be identified as one of the parents. We replicate our analyses in S4 Appendix excluding black respondents, and the results remain unchanged.

**Table 1:** Demographics by household type.

	Household Type		
	SS Female Couples	SS Male Couples	DS Couples
**Variables**	**Mean (*SE*)**	**Mean (*SE*)**	**Mean (*SE*)**
**Heterosexual**	0.81 (0.02)	0.69 (0.04)^a^	0.86 (0.001)^b^^,^^c^
**Female**	0.62 (0.02)	0.21 (0.04)^a^	0.54 (0.002)^b^^,c^
**First generation**	0.21 (0.02)	0.14 (0.03)^a^	0.16 (0.001)^b^
**Income**	6.84 (0.21)	9.79 (0.35)^a^	9.41 (0.011)^b^
**Age Group**	3.44 (0.04)	3.51 (0.09)	3.31 (0.002)^b^^,^^c^
**White**	0.34 (0.03)	0.52 (0.05)^a^	0.59 (0.002)^b^
**Black**	0.28 (0.02)	0.09 (0.03)^a^	0.08 (0.001)^b^
**Latino**	0.09 (0.01)	0.07 (0.03)	0.09 (0.001)
**Asian**	0.10 (0.02)	0.18 (0.03)^a^	0.10 (0.001)^c^
**Other**	0.19 (0.02)	0.14 (0.03)	0.14 (0.001)^b^
*N*	602	176	134,142

Weighted (unmatched) means and standard errors are reported. SS = same-sex; DS = different-sex.

^a^Difference between people with SS female parents and people with SS male parents is significant at *p* < .05 (one-tailed).

^b^Difference between people with SS female parents and people with DS parents is significant at *p* < .05 (one-tailed).

^c^Difference between people with SS male parents and people with DS parents is significant at *p* < .05 (one-tailed).

### Political attitudes

**Fig 1:**
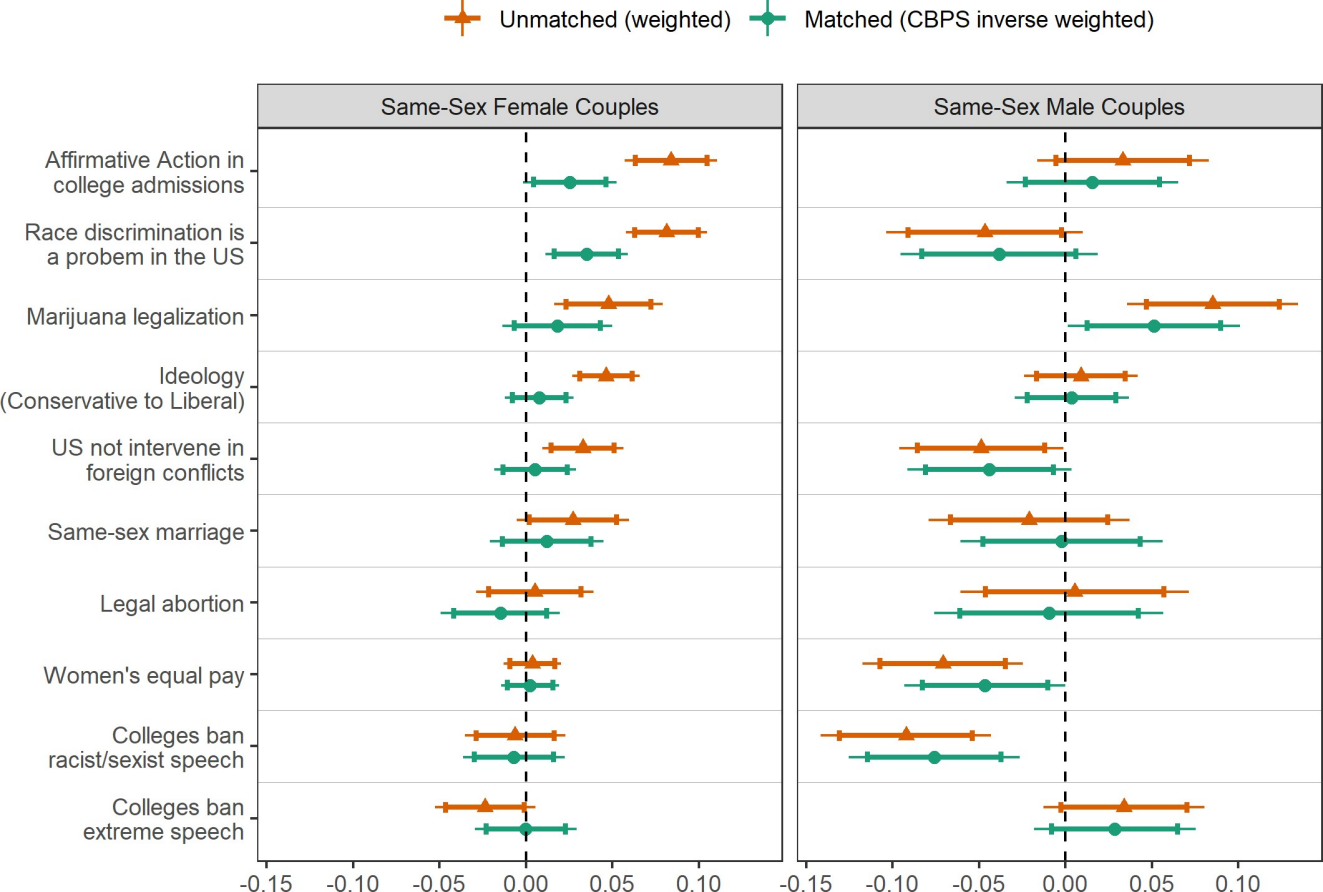
Differences in the political views of people with same-sex parents compared to those with different-sex parents. Positive differences indicate more progressive political attitudes; negative differences indicate less progressive political attitudes. 90% confidence intervals are represented by the error bars and 80% confidence intervals are represented by the cross-bars; *n*_min_ = 125,634 to *n*_max_ = 125,909 for SS female couple comparisons and *n*_min_ = 125,235 to *n*_max_ = 128,131 for SS male couple comparisons. Estimated differences are provided for people with DS parents relying on the HERI probability weights and also the CBPS inverse weights matching background characteristics of people with DS parents to people with SS parents.

Fig 1 provides differences in mean scores on political attitudes of people with SS parents and DS parents relying on unmatched and matched respondents. Positive differences suggest that people with SS parents are more progressive than people with DS parents, and negative differences suggest that people with SS parents are more conservative than people with DS parents. Prior to matching, people with SS female parents appear more socially progressive on affirmative action in college admissions, seeing race discrimination as a problem in the US, marijuana legalization, ideological self-placement, and US nonintervention into foreign conflicts. People with SS male parents appear more conservative on women’s workplace equality and colleges banning racist or sexist speech, and they are only more progressive on marijuana legalization. The average respondent to the Freshman Survey, however, is more left-of-center than the general public. After matching, the differences between people with SS parents and DS parents are substantially reduced. People with SS female parents hold more progressive views in affirmative action in college admissions and seeing racial discrimination as a problem. People with SS male parents hold more conservative attitudes about women’s equal pay; they are less likely to support colleges prohibiting racist or sexist speech and US nonintervention into foreign conflicts as compared to people with DS parents. People with SS male parents are only more progressive on marijuana legalization.

**Fig 2:**
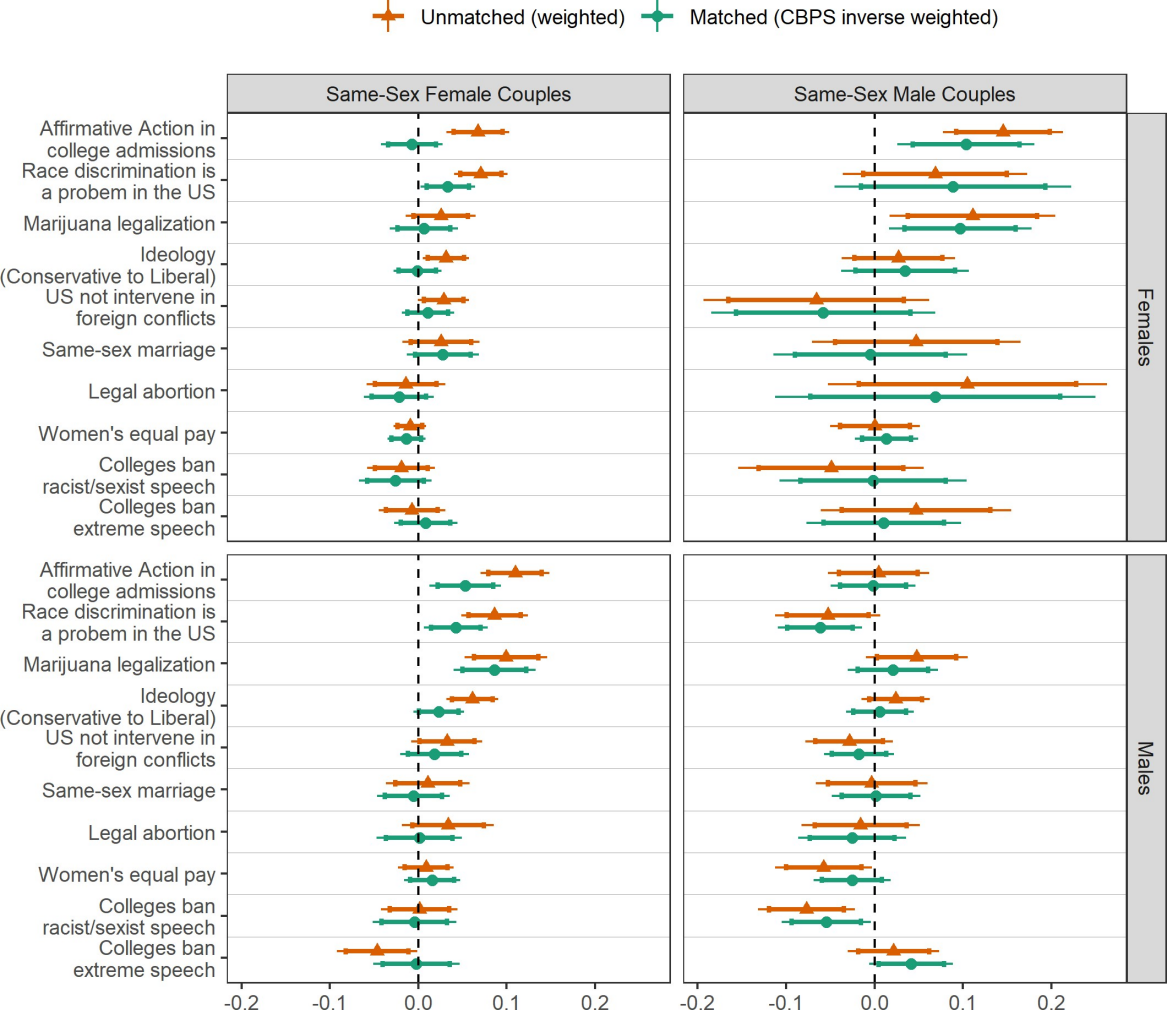
Differences in the political views of people with same-sex parents compared to those with different-sex parents, by sex. Positive differences indicate more progressive political attitudes; negative differences indicate less progressive political attitudes. 90% confidence intervals are represented by the error bars and 80% confidence intervals are represented by the cross-bars. Estimated differences are provided for people with DS parents relying on the HERI probability weights and also the CBPS inverse weights matching background characteristics of people with DS parents to people with SS parents.

Fig 2 provides differences between people with SS parents and DS parents by the respondent’s sex, relying on both the probability and final weights. Similar to Fig 1, positive differences suggest people with SS parents are more progressive than people with DS parents, and negative differences suggest they are more conservative. While there are few significant differences, we tend to observe that having SS female parents is associated with both more conservative and more progressive policy positions among women and more progressive policy positions among men when differences exist. Conversely, we observe that having SS male parents is associated with more conservative policy positions among men and more progressive policy positions among women. These patterns are consistent regardless of using the probability or the final weights.

#### Gender differences for people with same-sex female parents

Women who have SS female parents significantly differ from women who have DS parents in some of their political attitudes. Based on unmatched and unadjusted means, more women with SS female parents support affirmative action in college admissions, view race discrimination as a problem in the US, identify as more politically liberal, and support marriage equality than women with DS parents. While not statistically significant, women with SS female parents are slightly more conservative in their abortion attitudes, women’s equal pay, and whether colleges should ban racist or sexist speech. The only difference that remains significant after applying matching and adjustment is that women with SS female parents are more likely to view race discrimination as a problem in the US compared to women with DS parents. Men who have SS female parents significantly are more progressive than men who have DS parents in their attitudes about the use of affirmative action in college admissions, perceptions that race discrimination in the US, ideological self-placement, and opinions on marijuana legalization. These differences remain even after applying the final weights and adjustment. Men who have SS female parents were less likely to support colleges banning extremist speech, though this difference does not remain after weighting and adjustment.

#### Gender differences for people with same-sex male parents

Women who have SS male parents significantly differ from women who have DS parents in their attitudes on marijuana legalization and affirmative action in college admissions. These differences are present both prior to and after matching and regression adjustment. While not statistically significant, women with SS male parents tended to be more progressive in viewing race discrimination as a problem in the US and legal access to abortion. Men who have SS male parents significantly differ from men who have DS parents in their perceptions that race discrimination in the US, whether colleges should ban racist or sexist speech, and women’s equal pay in the workplace. Based on the unmatched and unadjusted estimates, men with SS male parents are more conservative in their attitudes on these topics. After matching and adjustment men with SS male parents remain more conservative in their views that race discrimination in a problem in the US and that colleges should ban racist or sexist speech, but there is not a significant difference in their views regarding women’s equal pay in the workplace.

## Discussion

What are the political attitudes of people with SS parents, and do they differ from people with DS parents? Prior to matching, people with SS parents are more progressive, except for respondents with SS male parents, who are more conservative on gender equality in the workplace. Differences diminish after matching, suggesting background characteristics that distinguish people with SS parents from people with DS parents likely lead to those differences. We do not find that people with SS female or male parents are much different from people DS parents. Indeed, on some measures people with SS male parents are more conservative than similarly situated people with DS parents. This is contrary to what is expected, and it fails to support our hypothesis. Given the exploratory nature of our study, future work should see if our findings replicate in other samples.

There are potentially a few reasons why we do not find support for our expectations. First, there may be differences in the political attitudes of LGB persons who choose to become parents as compared to LGB people in general. For example, in the cumulative General Social Survey, LGB parents are more likely to be political independents than LGB people who are not parents (see S5 Appendix). Thus, LGB parents may not hold as distinctly progressive political attitudes as LGB people in general. This may be particularly important to explain the somewhat more conservative attitudes of people with SS male parents. Second, our sample is of entering college first-years, and this age cohort may be distinctively more progressive on politics [e.g., 52] such that the distinctiveness we would expect to find by having SS parents is muted by an overall more progressive sample. Third, the sample size of people with SS parents is small relative to people with DS parents. While there is a large sample in the HERI survey data, these null patterns may be due to a lack of analytical power. If this is the case, then pooling multiple years of the HERI data may reveal patterns that are significant and distinct.

Our findings further suggest that there are meaningful differences between SS female and SS male households. People with SS female parents have lower household incomes and are more racially and ethnically diverse than people with SS male parents. SS male couples face, on average, greater costs to become parents, and SS female couples are more likely to be raising biological children [2]. Thus, socialization may be different in SS female households and SS male households, and this may intersect with both race and class. This is further supported by the demographic differences we observed in the data, as people with SS female parents are more likely to self-identify as black and have lower household incomes. The overall effects of SS parentage are being primarily driven by both the type of SS couple and the gender of the respondent. Men with same-sex male parents are more conservative, but men with same-sex female parents are more progressive. As prior work indicates that there are differences in the political socialization of boys and girls [34], we find divergences between males and females with SS parents. That most of these differences are among males complements prior work that suggests boys have a more malleable orientation toward politics than girls [33]. Thus, both the gendered nature of politics and gendered differences in political socialization can explain the distinctive patterns we observe for men with SS parents.

There are limitations in our current exploration. The secondary analysis of survey data means that we lack the ability to control the questionnaire design. This means that we have only one indicator of SS parentage. Thus, we lack an opportunity to more fully understand the households and characteristics of the respondents. Our analysis would be more thorough if we had measures of length of time in a SS parent household, aspects of family formation (e.g., if children were biological from a previous heterosexual relationship, if children were born through surrogacy; or if children were adopted), and other aspects of family stability. Prior research suggests the family formation SS households are distinct from DS households [3], so these measures may identify distinct subgroups of individuals with SS parents where their political attitudes are distinct (e.g., people who have had a long tenure of having SS parents with no family disruptions versus people who only recently had SS parents). Future primary data collection efforts can move beyond the current study’s limitations by incorporating such measures to evaluate political attitudes with a fuller understanding of the family formation process. Even with its limitations, our study at least can incorporate some characteristics of parents, which enables us to make more direct comparisons with similarly situated people with DS parents. Large-*N* studies of political attitudes rarely incorporate such details [14], even though prior work suggests that such details are important to understanding identity and attitude formation [14,32].

Despite these limitations, we present novel data to explore the potential for the distinct political attitudes of people with SS parents. Parental socialization is occasionally a factor in the political orientations of people with SS parents, though in unanticipated ways.

## Supporting information

S1 Appendix Variable names and question wordings. (DOCX)

S2 Appendix Propensity score estimation results. (DOCX)

S3 Appendix Regression results. (DOCX)

S4 Appendix Reanalyzing the data excluding black respondents. (DOCX)

S5 Appendix Partisanship in the general social survey. (DOCX)

S6 Appendix Unweighted results. (DOCX)
