# Supplementary material for: Potential differences between the political attitudes of people with same-sex parents and people with different-sex parents: An exploratory assessment of first-year college students
Source: PLoS One. 2021 Feb 25;16(2):e0246929. doi: 10.1371/journal.pone.0246929 (PMC7906383; doi:10.1371/journal.pone.0246929)
Supplement: S1 Appendix — (DOCX) [file pone.0246929.s001.docx]

**S1 Appendix. Variable Names and Question Wordings.**

Table S.1 contains the variables used in the matching and regression analyses in addition the question wordings. The full questionnaire is available at: <https://www.heri.ucla.edu/researchers/instruments/CIRP/2015-CIRP-Freshman-Survey.pdf>.

**Table S.1: Variable names and question wordings**

| **Variable** | **Wording** |
| --- | --- |
| Dependent variables: “Mark one in each row:” | |
| VIEW01 | “Racial discrimination is no longer a major problem in America.” |
| VIEW02 | “Abortion should be legal” |
| VIEW03 | “College have the right to ban extreme speakers from campus.” |
| VIEW05 | “Marijuana should be legalized.” |
| VIEW07 | “Colleges should prohibit racist/sexist speech on campus.” |
| VIEW08 | “The United States should intervene in the wars of other countries.” |
| VIEW09 | “Same-sex couples should have the right to legal marital status.” |
| VIEW10 | “Students from disadvantaged social background should be given preferential treatment in college admissions |
|  | Response set: “Agree Strongly”, “Agree Somewhat”, “Disagree Somehwat”, “Disagree Strongly” |
| POLIVIEW | “How would you characterize your political views?  Response set: “Far left”, “Liberal”, “Middle-of-the-road”, “Conservative”, “Far right” |
| Parent’s sex: “Please mark the sex of your parent(s) or guardian(s).” | |
| PARSEX1 | “Parent/Guardian 1” |
| PARSEX2 | “Parent/Guardian 2” |
|  | Response set: “Male”, “Female” |
| Covariates |  |
| SEX | “Your sex:”  Response set: “Male”, “Female” |
| AGE | “How old will you be on December 31 of this year? (Mark one)”  Response set: “16 or younger”, “17”, “18”, “19”, “20”, “21-24”, “25-29”, “30-39”, “40-54”, “55 or older”. |
| RACEGROUP | “Are you: (Mark all that apply)”  Response set: “White/Caucasian”, “African American/Black”, “American Indian/Alaska Native”, “East Asian (e.g., Chinese, Japanese, Korean, Taiwanese”, “Southeast Asian (e.g., Indian, Pakistani, Nepalese, Sri Lankan)”, “Other Asian”, “Native Hawaiian/Pacific Islander”, “Mexican American/Chicano”, “Puerto Rican”, “Other Latino”, “Other”.  *Note: variable is a recode from this question to: American Indian, Asian, Black, Hispanic, White, Other, Two or more race/ethnicity*. |
| LGBTQIDEN | “What is your sexual orientation?”  Response set: “Heterosexual/Straight”, “Gay”, “Lesbian”, “Bisexual”, “Queer”, “Other”. |
| PAREDUC1  PAREDUC2  FIRSTGEN | “What is the highest level of formal education obtained by your parents/guardians?”  Response set: “Junior high/Middle school or less”, “Some high school”, “High school graduate”, “Postsecondary school other than college”, “Some college”, “College degree”, “Some graduate school”, “Graduate degree”.  *Note: FIRSTGEN is a recode of respondents who have parent(s) who an educational attainment less than “some college.”* |
| INCOME | “What is your best estimate of your parents’/guardians’ total income last year? Consider income from all sources before taxes.”  Response set: “Less than $10,000”, “$10,000-14,999”, “$15,000-19,999”, “$20,000-24,999”, “$25,000-29,999”, “$30,000-39,999”, “$40,000-49,999”, “$50,000-$59,999”, “60,000-74,999”, “$75,000-99,999”, “$100,000-149,999”, “$150,000-199,999”, “$200,000-249,999”, “$250,000 or more”. |
| Additional matching covariates (used for propensity score estimation but not in the regression models) | |
| PARSTAT | “Are your parents: (Mark one):”  Response set: “Both alive and living with each other”, “Both alive, divorced or living apart”, “One or both deceased”. |
| PEMPLOY1  PEMPLOY2 | “Current employment status: (Mark one in each row):  Parent/Guardian 1  Parent/Guardian 2”  Response set: “Employed,” “Unemployed”, “Retired”. |
| SRELIGION  PRELIG1  PRELIG2 | “Current religious preference: (Mark one in each column)  Yours, Parent/Guardian 1, Parent/Guardian 2”  Response set: “Agnostics”, “Atheist”, “Baptist”, “Buddhist”, “Church of Christ”, “Eastern Orthodox”, “Episcopalian”, “Hindu”, “Jewish”, “LDS (Mormon)”, “Lutheran”, “Methodist”, “Muslim”, “Presbyterian”, “Quaker”, “Roman Catholic”, “Seventh-day Adventist”, “United Church of Christ/Congregational”, “Other Christian”, “Other Religion”, “None”.  Religion was coded to eight categories:   1. Christian 2. Catholic 3. Baptist 4. Agnostic/Atheist 5. Buddhist/Hindu 6. Muslim 7. Other 8. None |
