# Supplementary material for: Potential differences between the political attitudes of people with same-sex parents and people with different-sex parents: An exploratory assessment of first-year college students
Source: PLoS One. 2021 Feb 25;16(2):e0246929. doi: 10.1371/journal.pone.0246929 (PMC7906383; doi:10.1371/journal.pone.0246929)

# S2 Appendix. Propensity Score Estimation Results.

The results from the propensity score estimation are provided in Table S2.1. Notably, first-year college students are more likely to identify as lesbian, gay, bisexual, queer, or other than identify as heterosexual. Figure S2.1 provides a summary of balance before and after weighting on the propensity score. The propensity score estimation process provides test of model fit to determine whether the fitted model is inappropriate for the data. We find no indication that the model is mis-specified for SS female couples (Hansen’s-​*J* ​= * 10^−4)^ and for SS male couples (Hansen’s-​*J*​ = 1.98 * 10^−6^). Respondents who only have a single parent are removed from the analysis (N=6,269). Table S2.2 provides summary statistics for the respondents before and after weighting with the propensity score. Table S2.3 provides average estimates on political views, which is Figure 1 from the main text in a table format.

**Table S2.1: Covariate balance propensity score results**

|  | Same-Sex Female Parents | | | Same-Sex Male Parents |
| --- | --- | --- | --- | --- |
| *Gender (Ref. = Male)* |  | | |  |
| Female | 0.21 (0.06)*** | | | -1.47 (0.07)*** |
| *Age (Ref. = 16 or younger)* |  | | |  |
| 17 years old | -1.17 (0.49)* | | | 13.4 (1.1)*** |
| 18 years old | -0.63 (1.65) | | | 14 (3.96)*** |
| 19 years old | -0.36 (1.59) | | | 14 (3.84)*** |
| 20 years old | -0.01 (0.39) | | | 15 (0.93)*** |
| 21-24 years old | 0.37 (0.24) | | | 15.5 (0.54)*** |
| 25-29 years old | -14.2 (0.12)*** | | | 16.7 (0.2)*** |
| 30-39 years old | -14.8 (0.07)*** | | | 0.84 (0.2)*** |
| 40-54 years old | 2.42 (0.06)*** | | | -1.6 (0.15)*** |
| 55 or older | -13.5 (0.05)*** | | | -41.4 (0.1)*** |
| Age is missing | -0.28 (0.14)* | | | 15.7 (0.33)*** |
| *Parental status (Ref.= At least one parent is not alive)* |  | | |  |
| Parents divorced or living apart | -0.61 (0.27)* | -1.78 (0.62)** | | |
| Parents living together | -1.94 (0.28)*** | -1.24 (0.63). | | |
| Parental status is missing | -0.59 (0.24)* | 0.34 (0.12)** | | |
| *Educational attainment of parents (Ref. = Both have BA degrees)* |  |  | | |
| BA – Post-grad | 0.13 (0.07)* | 0.11 (0.08) | | |
| BA – HS | -0.29 (0.2) | 0.74 (0.1)*** | | |
| BA – Less HS | -0.14 (0.05)** | -13.8 (0.08)*** | | |
| BA - Unknown | 1.71 (0.08)*** | 1.73 (0.12)*** | | |
| BA – Trade School | -0.89 (0.06)*** | 0.36 (0.13)** | | |
| BA – Some college | -0.29 (0.11)** | -0.14 (0.11) | | |
| BA – Some Graduate School | -0.86 (0.06)*** | 0.61 (0.12)*** | | |
| BA – Some high school | -0.3 (0.07)*** | 0.62 (0.09)*** | | |
| Post-grad – Post-grad` | 0.44 (0.1)*** | 0.87 (0.12)*** | | |
| Post-grad – HS | -0.55 (0.08)*** | -0.66 (0.16)*** | | |
| Post-grad – Less HS | 0.61 (0.06)*** | 1.36 (0.14)*** | | |
| Post-grad - Unknown | 1.44 (0.06)*** | -14 (0.1)*** | | |
| Post-grad – Trade School | -1.13 (0.08)*** | -0.21 (0.19) | | |
| Post-grad – Some college | 0.14 (0.26) | -0.28 (0.1)** | | |
| Post-grad – Some graduate school | 0.27 (0.11)* | 0.26 (0.16) | | |
| Post-Grad – Some high school | -0.62 (0.14)*** | -13.6 (0.1)*** | | |
| HS - HS | -0.17 (0.09). | 0.74 (0.18)*** | | |
| HS – Less HS | 0.03 (0.19) | -12.8 (0.12)*** | | |
| HS - Unknown | 1.1 (0.05)*** | 1.78 (0.14)*** | | |
| HS – Trade School | 0.04 (0.06) | 1.95 (0.15)*** | | |
| HS – Some college | -0.19 (0.07)** | 0.4 (0.1)*** | | |
| HS – Some graduate school | -13.2 (0.06)*** | -13.7 (0.08)*** | | |
| HS – Some high school | -0.1 (0.06) | 0.85 (0.17)*** | | |
| Less HS – Less HS | -0.16 (0.08)* | 0.67 (0.18)*** | | |
| Less HS - Unknown | -14 (0.08)*** | -13.4 (0.08)*** | | |
| Less HS – Trade School | 0.09 (0.06) | -12.7 (0.11)*** | | |
| Less HS – Some college | -0.48 (0.06)*** | -13.5 (0.07)*** | | |
| Less HS – Some graduate school | -13.2 (0.08)*** | -13.6 (0.1)*** | | |
| Less HS – Some high school | -0.59 (0.13)*** | 0.49 (0.2)* | | |
| Unknown – Unknown | -0.12 (0.05)** | 0.04 (0.07) | | |
| Unknown – Trade School | 1.91 (0.14)*** | 2.92 (0.11)*** | | |
| Unknown – Some college | 1.02 (0.05)*** | -13.4 (0.1)*** | | |
| Unknown – Some graduate school | 1.36 (0.14)*** | -13.6 (0.11)*** | | |
| Unknown – Some high school | 1.38 (0.06)*** | -13.2 (0.16)*** | | |
| Trade School – Trade School | -0.59 (0.13)*** | -12.8 (0.12)*** | | |
| Trade School – Some college | -0.21 (0.15) | -0.96 (0.07)*** | | |
| Trade School – Some graduate school | 0.18 (0.13) | -14 (0.08)*** | | |
| Trade School – Some high school | 0.23 (0.08)** | -12.7 (0.07)*** | | |
| Some College – Some college | 0.15 (0.11) | -0.05 (0.08) | | |
| Some College – Some graduate school | -0.25 (0.18) | -13.8 (0.08)*** | | |
| Some College – Some high school | -0.99 (0.11) | -13.6 (0.1)*** | | |
| Some high school – some high school | 1.05 (0.07) | 2.38 (0.11)*** | | |
| *Employment status of parents (Ref. = Both Employed* |  |  | | |
| One employed – one unknown | -0.01 (0.06) | -0.05 (0.11) | | |
| One employed – one retired | 1.1 (0.06)*** | -1.22 (0.09)*** | | |
| One employed – one unemployed | -0.14 (0.08)^ | -0.1 (0.06) | | |
| Both unknown | 0.44 (0.18)* | -0.33 (0.07)*** | | |
| One unknown – one retired | -1.4 (0.09)*** | -15.1 (0.28)*** | | |
| One unknown – one unemployed | 0.02 (0.08) | -14.6 (0.15)*** | | |
| Both retired | 0.22 (0.09)* | 0.05 (0.07) | | |
| One retired- one unemployed | 0.69 (0.08)*** | 0.11 (0.13) | | |
| Both unemployed | 0.69 (0.07)*** | 0.87 (0.09)*** | | |
| *Household Income (Ref. = Less than $10,000)* |  |  | | |
| $10,000-$14,999 | 0.15 (0.1) | 0.04 (0.1) | | |
| $15,000-$19,999 | 0.05 (0.11) | -0.11 (0.11) | | |
| $20,000-$24,999 | -0.2 (0.11)^ | -2.46 (0.13)*** | | |
| $25,000-$29,999 | -0.22 (0.11)* | -0.42 (0.12)*** | | |
| $30,000-$39,999 | -0.2 (0.12) | -1.92 (0.14)*** | | |
| $40,000-$49,999 | -0.45 (0.13)*** | -0.3 (0.15)* | | |
| $50,000-$59,999 | -0.67 (0.14)*** | -0.13 (0.2) | | |
| $60,000-$74,999 | -0.49 (0.14)*** | -0.71 (0.18)*** | | |
| $75,000-$99,999 | -0.67 (0.17)*** | -0.38 (0.18)* | | |
| $100,000-$149,999 | -1.19 (0.19)*** | -0.16 (0.22) | | |
| $150,000-$199,999 | -0.69 (0.16)*** | -0.25 (0.17) | | |
| $200,000-$249,999 | -1.38 (0.13)*** | 0.04 (0.17) | | |
| $250,000 or more | -1.16 (0.18)*** | -0.19 (0.2) | | |
| Income is missing | -0.65 (0.17)*** | -0.33 (0.16)* | | |
| *Religious Affiliation of parents (Ref. = Both Atheist or Agnostic)* |  |  | | |
| One atheist/agnostic – One Baptist | -0.41 (0.19)* | 0.49 (0.07)*** | | |
| One atheist/agnostic – One Catholic | 2.25 (0.06)*** | -13.5 (0.17)*** | | |
| One atheist/agnostic – One Christian | -0.08 (0.12) | -0.86 (0.12)*** | | |
| One atheist/agnostic – One Hindu or Buddhist | 0.74 (0.07)*** | -0.16 (0.11) | | |
| One atheist/agnostic – One Jewish | 0.5 (0.09)*** | 0.17 (0.08)* | | |
| One atheist/agnostic – One Muslim | 0.37 (0.06)*** | -13.4 (0.07)*** | | |
| One atheist/agnostic – One Unknown | 0.09 (0.07) | -14.3 (0.08)*** | | |
| One atheist/agnostic – One Other | 0.04 (0.08) | 1.23 (0.11)*** | | |
| Both Baptist | -0.4 (0.18)* | 0.35 (0.2). | | |
| One Baptist – One Catholic | -13.2 (0.06)*** | -13.9 (0.08)*** | | |
| One Baptist – One Christian | -0.54 (0.1)*** | -1.61 (0.1)*** | | |
| One Baptist – One Hindu or Buddhist | -14.1 (0.06)*** | -14 (0.08)*** | | |
| One Baptist – One Jewish | 1.71 (0.05)*** | -14 (0.11)*** | | |
| One Baptist – One Muslim | -13.7 (0.07)*** | -13.3 (0.09)*** | | |
| One Baptist – One Unknown | -0.38 (0.05)*** | -14.1 (0.09)*** | | |
| One Baptist – One Other | -0.59 (0.05)*** | -13.7 (0.08)*** | | |
| Both Catholic | 0.32 (0.04)*** | -13.4 (0.09)*** | | |
| One Catholic – One Christian | -0.32 (0.1)** | 0.56 (0.1)*** | | |
| One Catholic – One Hindu or Buddhist | -13.4 (0.09)*** | -14.8 (0.22)*** | | |
| One Catholic – One Jewish | -13 (0.09)*** | -13.7 (0.09)*** | | |
| One Catholic – One Muslim | -12.5 (0.05)*** | -13.1 (0.06)*** | | |
| One Catholic – One Unknown | -14 (0.05)*** | 4.22 (0.13)*** | | |
| One Catholic – One Other | -12.2 (0.06)*** | -12.3 (0.07)*** | | |
| Both Christian | -0.46 (0.16)** | 0.37 (0.17)* | | |
| One Christian – One Hindu or Buddhist | -0.34 (0.07)*** | -1.27 (0.09)*** | | |
| One Christian – One Jewish | -0.16 (0.08)* | 0.42 (0.11)*** | | |
| One Christian – One Muslim | -0.33 (0.09)*** | -13.4 (0.08)*** | | |
| One Christian – One Unknown | -0.48 (0.06)*** | 0.17 (0.08)* | | |
| One Christian – One Other | -0.66 (0.06)*** | -13.5 (0.08)*** | | |
| Both Hindu/Buddhist | -0.7 (0.14)*** | -0.66 (0.1)*** | | |
| One Hindu/Buddhist – One Jewish | 0.84 (0.1)*** | -13.9 (0.09)*** | | |
| One Hindu/Buddhist – One Muslim | -13.6 (0.12)*** | -14.6 (0.07)*** | | |
| One Hindu/Buddhist – One Unknown | -0.2 (0.07)** | -15.1 (0.14)*** | | |
| One Hindu/Buddhist – One Other | -0.31 (0.05)*** | -14.4 (0.11)*** | | |
| Both Jewish | 0.47 (0.23)* | 0.61 (0.29)* | | |
| One Jewish – One Muslim | -12.7 (0.15)*** | -13.4 (0.07)*** | | |
| One Jewish – One Unknown | 1.09 (0.07)*** | -13.1 (0.11)*** | | |
| One Jewish – One Other | 1.44 (0.16)*** | -13.9 (0.14)*** | | |
| Both Muslim | -0.01 (0.1) | 0.57 (0.16)*** | | |
| One Muslim – One Unknown | 0.49 (0.1)*** | -13.1 (0.11)*** | | |
| One Muslim – One Other | -13.5 (0.24)*** | -13 (0.1)*** | | |
| Both Unknown | -0.14 (0.09) | 0.6 (0.08)*** | | |
| One unknown – One other | 0.36 (0.07)*** | -13.8 (0.12)*** | | |
| Both Other | -0.53 (0.11)*** | 0.81 (0.16)*** | | |
| *Self Religious Affiliation (Ref. = Atheist/Agnostic)* |  | |  | |
| Baptist | -0.12 (0.17) | | 0.43 (0.2)* | |
| Catholic | 0.04 (0.03) | | -1.19 (0.02)*** | |
| Christian | -0.12 (0.13) | | -0.42 (0.09)*** | |
| Hindu or Buddhist | 0.3 (0.14)* | | 0.64 (0.07)*** | |
| Jewish | -0.1 (0.24) | | -1.51 (0.28)*** | |
| Muslim | -0.49 (0.1)*** | | -1.33 (0.15)*** | |
| Self religion is missing | 0.2 (0.08)** | | -0.56 (0.08)*** | |
| Other religion | 0.59 (0.11)*** | | -1.75 (0.15)*** | |
| *First Generation (Ref.* = *Yes*) |  | |  | |
| No | -0.23 (0.17) | | -0.89 (0.19)*** | |
| First Generation is Missing | -0.12 (0.05)** | | 0.04 (0.07) | |
| *LGBQ Identification (Ref. = Heterosexual)* |  | |  | |
| Gay | -0.96 (0.08)*** | | 0.99 (0.08)*** | |
| Lesbian | 0.66 (0.07)*** | | -12.9 (0.14)*** | |
| Bisexual | 0.25 (0.06)*** | | 1.3 (0.09)*** | |
| Queer | 0.11 (0.07) | | 2.31 (0.12)*** | |
| Other | 0.16 (0.06)* | | 1.47 (0.1)*** | |
| LGBQ Identification is Missing | -0.02 (0.06) | | 0.76 (0.07)*** | |
| *Race/Ethnicity (Ref. = American Indian* |  | |  | |
| Asian | 1.1 (0.86) | | 1.17 (0.77) | |
| Black | 1.69 (0.66)* | | 0.61 (0.6) | |
| Hispanic/Latino | 0.84 (0.64) | | 0.63 (0.61) | |
| White | 0.5 (1.21) | | 0.48 (1.15) | |
| Other | 0.6 (0.27)* | | 1.44 (0.23)*** | |
| Multiracial | 1.12 (0.77) | | 0.56 (0.73) | |
| Race/ethnicity is missing | 1.05 (0.37)** | | 1.12 (0.37)** | |
| Intercept | -3.89 (4.34) | | -19.6 (9.16)* | |
| *N* | 134,744 | | 134,318 | |
| Log-likelihood | -3125.67 | | -1,217.01 | |
| Hansen’s-​*J* | 5.75 * 10^−8^ | | 1.11 * 10^−6^ | |

*Note:* ​^*p* < 0.10; **p* < 0.05; ***p* < 0.01; ****p* < 0.001 (two-tailed).

Table S2.2. Demographic Characteristics by Household Type after Matching

|  | **Same-sex female couples** | |  | **Same-sex male couples** | |  |
| --- | --- | --- | --- | --- | --- | --- |
|  | **Same-sex** | **Different-sex** | | **Same-sex** | **Different-sex** | |
| **Variable** | **Mean (SE)** | **Mean (SE)** | | **Mean (SE)** | **Mean (SE)** | |
| **Heterosexual** | 0.81 (0.02) | 0.81 (0.004) | | 0.69 (0.04) | 0.70 (0.004) | |
| **Female** | 0.62 (0.02) | 0.62 (0.004) | | 0.21 (0.05) | 0.21 (0.003) | |
| **First generation** | 0.21 (0.02) | 0.21 (0.004) | | 0.14 (0.04) | 0.13 (0.003) | |
| **Income** | 6.84 (0.21) | 6.84 (0.04) | | 9.79 (0.35) | 9.77 (0.04) | |
| **Age** | 3.44 (0.04) | 3.44 (0.01) | | 3.51 (0.09) | 3.48 (0.01) | |
| **White** | 0.34 (0.03) | 0.34 (0.004) | | 0.52 (0.05) | 0.52 (0.004) | |
| **Black** | 0.28 (0.02) | 0.28 (0.005) | | 0.09 (0.03) | 0.09 (0.003) | |
| **Latino** | 0.09 (0.01) | 0.09 (0.002) | | 0.07 (0.03) | 0.07 (0.002) | |
| **Asian** | 0.10 (0.02) | 0.10 (0.002) | | 0.18 (0.04) | 0.18 (0.003) | |
| **Other** | 0.19 (0.02) | 0.19 (0.003) | | 0.14 (0.03) | 0.14 (0.003) | |

Note: Same-sex female couples ​*N=602*​; same-sex male couples ​*N =* ​176, comparison sample ​*N* ​= 134,142; means are reported; standard errors are in the parentheses; significance tests are for differences between children of same-sex couples and children of different-sex couples; *p<0.05 (one-tailed).

Table S2.3: Political views

|  | Same-sex female couples | | | | Same-sex male couples | | |  |
| --- | --- | --- | --- | --- | --- | --- | --- | --- |
|  | Unmatched (weighted) | | Matched |  | Unmatched (weighted) | | Matched |  |
|  | Same- sex | Different- sex | Different- sex | *N* | Same- sex | Different- sex | Different- sex | *N* |
| Ideology  (Conservative to Liberal) | .58  (.01) | .53  (.0008)* | .57  (.002) | 125,909 | .54  (.02) | .53  (.001) | .54  (.002) | 125,517 |
| Racial discrimination is a problem in the US | .82  (.01) | .73  (.001)* | .78  (.002)* | 128,542 | .69  (.03) | .73  (.001) | .73  (.002) | 128,131 |
| Abortion should be legal | .60  (0.02) | .59  (.001) | .61  (.003) | 127,763 | .60  (.04) | .59  (.001) | .61  (.003) | 127,356 |
| Colleges should ban extreme speech | .42  (.02) | .45  (.001) | .42  (.003) | 127,334 | .48  (.03) | .45  (.001) | .45  (.003) | 126,933 |
| Marijuana legalization | .57  (.02) | .52  (.001)* | .55  (.003) | 127,274 | .61  (.03) | .52  (.001)* | .56  (.003)* | 126,868 |
| College should prohibit  racist/sexist speech | .67  (.02) | .67  (.001) | .67  (.003) | 126,343 | .58  (.03) | .67  (.001)* | .66  (.003)* | 125,936 |
| Gender workplace equality | .92  (.01) | .91  (.0007) | .92  (.002) | 126,854 | .84  (.03) | .91  (.001)* | .89  (.002) | 126,453 |
| US should not intervene in conflicts | .67 (.01) | .64  (.001)* | .66  (.003) | 126,067 | .59 (.03) | .64  (.001)* | .63  (.002) | 125,668 |
| Same-sex marriage | .81  (.02) | .78  (.001) | .79  (.003) | 126,334 | .76  (.04) | .78  (.001) | .76  (.003) | 125,932 |
| Affirmative action in college admissions | .58 (.02) | .49  (.001)* | .55  (.003) | 125,634 | .53 (.03) | .49  (.001) | .51  (.003) | 125,235 |

Note: All variables scaled from zero to one with higher values indicating more progressive responses. Differences between people with same-sex parents and different-sex parents; standard errors are in the parentheses; *p<0.05 (one-tailed).

Table S2.3: Political views among females

|  | Same-sex female couples | | | | Same-sex male couples | | |  |
| --- | --- | --- | --- | --- | --- | --- | --- | --- |
|  | Unmatched (weighted) | | Matched |  | Unmatched (weighted) | | Matched |  |
|  | Same- sex | Different- sex | Different- sex | *N* | Same- sex | Different- sex | Different- sex | *N* |
| Ideology  (Conservative to Liberal) | .59  (.02) | .56  (.001)* | .59  (.002) | 125,909 | .58  (.04) | .56  (.001) | .59  (.003) | 125,517 |
| Racial discrimination is a problem in the US | .83  (.02) | .76  (.001)* | .81  (.003) | 128,542 | .83  (.06) | .76  (.001) | .78  (.004) | 128,131 |
| Abortion should be legal | .58  (0.03) | .60  (.002) | .62  (.005) | 127,763 | .70  (.10) | .60  (.002) | .63  (.006) | 127,356 |
| Colleges should ban extreme speech | .43  (.02) | .44  (.001) | .42  (.003) | 127,334 | .49  (.07) | .44  (.001) | .43  (.004) | 126,933 |
| Marijuana legalization | .52  (.02) | .49  (.002) | .54  (.004) | 127,274 | .60  (.06) | .49  (.001)* | .52  (.005) | 126,868 |
| College should prohibit  racist/sexist speech | .68  (.02) | .70  (.002) | .70  (.004) | 126,343 | .65  (.06) | .70  (.002) | .71  (.006) | 125,936 |
| Gender workplace equality | .94  (.01) | .95  (.0009) | .94  (.003) | 126,854 | .95  (.03) | .87  (.001)* | .95  (.003) | 126,453 |
| US should not intervene in conflicts | .68 (.02) | .65  (.001) | .68  (.004) | 126,067 | .59 (.08) | .65  (.001) | .66  (.004) | 125,668 |
| Same-sex marriage | .84  (.03) | .82  (.002) | .84  (.003) | 126,334 | .87  (.07) | .82  (.002) | .83  (.004) | 125,932 |
| Affirmative action in college admissions | .60 (.02) | .50  (.002)* | .56  (.004) | 125,634 | .64 (.04) | .50  (.002)* | .51  (.003)* | 125,235 |

Note: All variables scaled from zero to one with higher values indicating more progressive responses. Differences between people with same-sex parents and different-sex parents; standard errors are in the parentheses; *p<0.05 (one-tailed).

Table S2.3: Political views among males

|  | Same-sex female couples | | | | Same-sex male couples | | |  |
| --- | --- | --- | --- | --- | --- | --- | --- | --- |
|  | Unmatched (weighted) | | Matched |  | Unmatched (weighted) | | Matched |  |
|  | Same- sex | Different- sex | Different- sex | *N* | Same- sex | Different- sex | Different- sex | *N* |
| Ideology  (Conservative to Liberal) | .57  (.02) | .51  (.001)* | .54  (.003)* | 125,909 | .53  (.02) | .51 (.001) | .53  (.002) | 125,517 |
| Racial discrimination is a problem in the US | .79  (.02) | .70  (.001)* | .74  (.004)* | 128,542 | .65  (.04) | .70  (.001) | .71  (.003)* | 128,131 |
| Abortion should be legal | .61  (0.03) | .58  (.002) | .60  (.005) | 127,763 | .56  (.04) | .58  (.002) | .60  (.004) | 127,356 |
| Colleges should ban extreme speech | .41  (.03) | .46  (.001)* | .43  (.005) | 127,334 | .48  (.03) | .46  (.002) | .46  (.003) | 126,933 |
| Marijuana legalization | .66  (.03) | .56  (.002)* | .59  (.005)* | 127,274 | .61  (.03) | .56  (.002) | .57  (.004) | 126,868 |
| College should prohibit  racist/sexist speech | .64  (.03) | .64  (.002) | .63  (.005) | 126,343 | .56  (.03) | .64  (.002)* | .64 (.003)* | 125,936 |
| Gender workplace equality | .88  (.02) | .87  (.001) | .87  (.003) | 126,854 | .81  (.03) | .87  (.001)* | .87  (.002)* | 126,453 |
| US should not intervene in conflicts | .65 (.02) | .62  (.001) | .64  (.004) | 126,067 | .59  (.03) | .62  (.001)* | .62  (.003) | 125,668 |
| Same-sex marriage | .74  (.02) | .73  (.002) | .73  (.006) | 126,334 | .73  (.04) | .73  (.002) | .74  (.003) | 125,932 |
| Affirmative action in college admissions | .60  (.02) | .49  (.001)* | .55  (.005)* | 125,634 | .50 (.03) | .49  (.001) | .51  (.003) | 125,235 |

Note: All variables scaled from zero to one with higher values indicating more progressive responses. Differences between people with same-sex parents and different-sex parents; standard errors are in the parentheses; *p<0.05 (one-tailed).

**Figure S2.1: Standardized mean differences in before-and-after propensity score estimation**

Same-Sex Male Couples

Same-Sex Female Couples


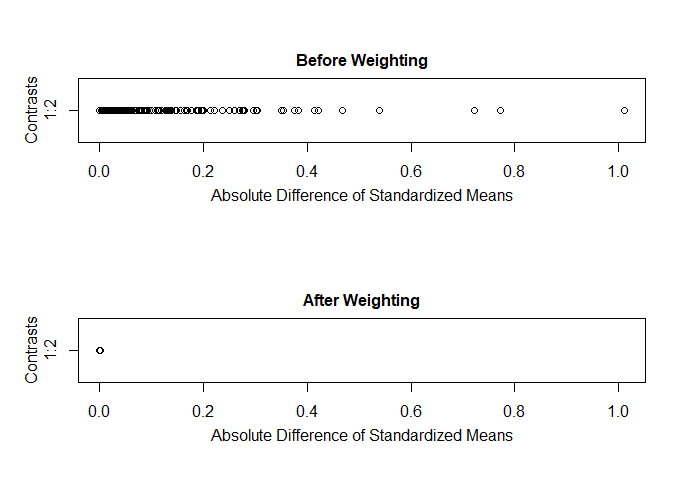

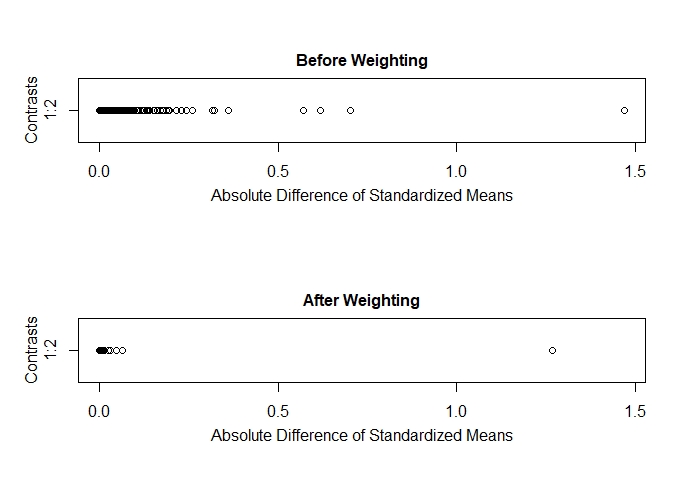

Supplement: S2 Appendix — (DOCX) [file pone.0246929.s002.docx]
