# Supplementary material for: Potential differences between the political attitudes of people with same-sex parents and people with different-sex parents: An exploratory assessment of first-year college students
Source: PLoS One. 2021 Feb 25;16(2):e0246929. doi: 10.1371/journal.pone.0246929 (PMC7906383; doi:10.1371/journal.pone.0246929)
Supplement: S3 Appendix — (DOCX) [file pone.0246929.s003.docx]

**S3 Appendix. Regression Results**

The main text reports mean scores on the political attitudes variables and predicted values from OLS regression models interacting respondent’s sex with whether or not they have same-sex parents. Regressions were relied upon because they increased the efficiency of the estimates, revealing more significant differences than if confounders were not modeled. Table S2.3 provides mean differences between people with SS parents and DS parents broken-down by gender. While point estimates are similar, the confidence intervals are tighter with model predictions. The regression results are provided in Tables S3.1-S3.5. The complex design of the survey is considered for point estimates standard error estimates. These models control for age, race or ethnicity, household income, LGBT identification, and first-generation status. The only difference between reporting mean scores in the main text versus the regression results is that the difference between people with SS male parents and DS parents in their attitudes about US intervention into foreign conflicts is no longer significant. All other patterns are consistent with what is reported in the main text.

| **Table S3.1: Regression Results, Same-Sex Female Couples** | | | | | | | | | | | | | | | | | | | | |
| --- | --- | --- | --- | --- | --- | --- | --- | --- | --- | --- | --- | --- | --- | --- | --- | --- | --- | --- | --- | --- |
|  | (1) | | (2) | (3) | | | | (4) | (5) | | | (6) | | (7) | | (8) | (9) | | (10) | |
|  | Ideology | | Ideology | Racism in US | | | | Racism in US | Abortion | | | Abortion | | Coll. Ban Ext. Speech | | Coll. Ban Ext. Speech | Marijuana | | Marijuana | |
| Same-Sex Couple | 0.0084 | | 0.023^ | 0.037** | | | | 0.042* | -0.013 | | | 0.0011 | | 0.0043 | | -0.0022 | 0.037* | | 0.086** | |
|  | (0.012) | | (0.018) | (0.014) | | | | (0.022) | (0.019) | | | (0.029) | | (0.018) | | (0.030) | (0.018) | | (0.028) | |
| Female | 0.030* | | 0.042*** | 0.045** | | | | 0.050*** | -0.031^ | | | -0.019* | | 0.0018 | | -0.0036 | -0.12*** | | -0.079*** | |
|  | (0.013) | | (0.0054) | (0.016) | | | | (0.0072) | (0.020) | | | (0.0085) | | (0.020) | | (0.0080) | (0.019) | | (0.0081) | |
| Asian | 0.12*** | | 0.12*** | 0.22^ | | | | 0.22^ | -0.0020 | | | -0.0038 | | 0.065 | | 0.065 | -0.35* | | -0.36* | |
|  | (0.036) | | (0.036) | (0.14) | | | | (0.14) | (0.053) | | | (0.055) | | (0.056) | | (0.057) | (0.16) | | (0.16) | |
| Black | 0.12*** | | 0.12*** | 0.35** | | | | 0.35** | -0.16*** | | | -0.16*** | | -0.0034 | | -0.0022 | -0.21^ | | -0.22^ | |
|  | (0.035) | | (0.035) | (0.13) | | | | (0.13) | (0.051) | | | (0.052) | | (0.056) | | (0.057) | (0.15) | | (0.16) | |
| Hispanic | 0.14*** | | 0.14*** | 0.25* | | | | 0.25* | -0.12* | | | -0.13* | | 0.019 | | 0.020 | -0.29* | | -0.30* | |
|  | (0.037) | | (0.037) | (0.14) | | | | (0.14) | (0.060) | | | (0.061) | | (0.059) | | (0.060) | (0.15) | | (0.16) | |
| White | 0.11** | | 0.11** | 0.25* | | | | 0.25* | -0.046 | | | -0.048 | | 0.043 | | 0.044 | -0.25^ | | -0.26^ | |
|  | (0.036) | | (0.036) | (0.13) | | | | (0.13) | (0.050) | | | (0.051) | | (0.056) | | (0.057) | (0.15) | | (0.16) | |
| Other | 0.11** | | 0.11** | 0.00038 | | | | -0.00029 | -0.0100 | | | -0.012 | | 0.063 | | 0.064 | -0.13 | | -0.14 | |
|  | (0.036) | | (0.035) | (0.19) | | | | (0.19) | (0.13) | | | (0.14) | | (0.088) | | (0.088) | (0.19) | | (0.20) | |
| Multi-racial | 0.13*** | | 0.13*** | 0.29* | | | | 0.29* | -0.097* | | | -0.100* | | -0.0017 | | -0.00023 | -0.25^ | | -0.26^ | |
|  | (0.036) | | (0.036) | (0.13) | | | | (0.13) | (0.055) | | | (0.056) | | (0.059) | | (0.060) | (0.15) | | (0.16) | |
| Gay | 0.11** | | 0.11** | 0.096** | | | | 0.096** | 0.056 | | | 0.055 | | 0.051 | | 0.051 | -0.057 | | -0.060 | |
|  | (0.041) | | (0.041) | (0.035) | | | | (0.035) | (0.047) | | | (0.047) | | (0.045) | | (0.046) | (0.058) | | (0.060) | |
| Lesbian | 0.074* | | 0.073* | 0.045* | | | | 0.045* | 0.100* | | | 0.099* | | 0.016 | | 0.016 | 0.15** | | 0.15** | |
|  | (0.039) | | (0.040) | (0.026) | | | | (0.025) | (0.048) | | | (0.048) | | (0.049) | | (0.049) | (0.052) | | (0.051) | |
| Bisexual | 0.11*** | | 0.11*** | -0.011 | | | | -0.011 | 0.14*** | | | 0.14*** | | -0.00040 | | -0.00041 | 0.076* | | 0.076* | |
|  | (0.024) | | (0.024) | (0.029) | | | | (0.029) | (0.025) | | | (0.026) | | (0.028) | | (0.028) | (0.035) | | (0.035) | |
| Queer | 0.083 | | 0.082 | -0.22* | | | | -0.22* | 0.019 | | | 0.018 | | 0.19*** | | 0.19*** | -0.082 | | -0.087 | |
|  | (0.12) | | (0.12) | (0.12) | | | | (0.12) | (0.096) | | | (0.096) | | (0.060) | | (0.061) | (0.13) | | (0.13) | |
| Other | 0.057^ | | 0.057^ | 0.085** | | | | 0.085** | 0.18*** | | | 0.18*** | | -0.067^ | | -0.067^ | 0.10* | | 0.10* | |
|  | (0.035) | | (0.035) | (0.029) | | | | (0.028) | (0.041) | | | (0.041) | | (0.052) | | (0.052) | (0.046) | | (0.045) | |
| First Gen | -0.030* | | -0.030* | -0.051* | | | | -0.051* | 0.0059 | | | 0.0057 | | 0.0067 | | 0.0068 | 0.0080 | | 0.0075 | |
|  | (0.014) | | (0.014) | (0.025) | | | | (0.025) | (0.025) | | | (0.025) | | (0.023) | | (0.023) | (0.023) | | (0.023) | |
| $10,000 to 14,999 | 0.0018 | | 0.00052 | -0.012 | | | | -0.012 | -0.047 | | | -0.048 | | -0.022 | | -0.021 | 0.047 | | 0.043 | |
|  | (0.020) | | (0.020) | (0.042) | | | | (0.042) | (0.046) | | | (0.046) | | (0.037) | | (0.037) | (0.038) | | (0.038) | |
| $15,000 to 19,999 | -0.00084 | | -0.0014 | 0.034 | | | | 0.034 | -0.059^ | | | -0.060^ | | -0.013 | | -0.013 | 0.013 | | 0.011 | |
|  | (0.027) | | (0.027) | (0.034) | | | | (0.034) | (0.045) | | | (0.045) | | (0.039) | | (0.039) | (0.052) | | (0.053) | |
| $20,000 to 24,999 | 0.036* | | 0.036* | 0.0012 | | | | 0.0012 | -0.033 | | | -0.033 | | -0.015 | | -0.015 | 0.055^ | | 0.054^ | |
|  | (0.021) | | (0.022) | (0.035) | | | | (0.035) | (0.050) | | | (0.050) | | (0.038) | | (0.038) | (0.040) | | (0.040) | |
| $25,000 to 29,999 | 0.0024 | | 0.0022 | 0.058* | | | | 0.058* | 0.00018 | | | -0.000014 | | 0.040 | | 0.040 | -0.0090 | | -0.0095 | |
|  | (0.029) | | (0.030) | (0.032) | | | | (0.032) | (0.048) | | | (0.048) | | (0.051) | | (0.052) | (0.057) | | (0.056) | |
| $30,000 to 39,999 | 0.021 | | 0.021 | 0.081** | | | | 0.081** | 0.022 | | | 0.021 | | -0.000098 | | 0.000026 | 0.063* | | 0.063* | |
|  | (0.021) | | (0.021) | (0.029) | | | | (0.029) | (0.039) | | | (0.039) | | (0.035) | | (0.035) | (0.036) | | (0.036) | |
| $40,000 to 49,999 | 0.0050 | | 0.0049 | -0.0079 | | | | -0.0080 | -0.0091 | | | -0.0092 | | -0.023 | | -0.023 | 0.028 | | 0.027 | |
|  | (0.023) | | (0.023) | (0.047) | | | | (0.047) | (0.037) | | | (0.037) | | (0.042) | | (0.042) | (0.041) | | (0.041) | |
| $50,000 to 59,999 | 0.049^ | | 0.049^ | 0.047 | | | | 0.047 | 0.000059 | | | 0.000098 | | -0.052^ | | -0.052^ | 0.023 | | 0.024 | |
|  | (0.032) | | (0.033) | (0.038) | | | | (0.038) | (0.051) | | | (0.051) | | (0.035) | | (0.035) | (0.060) | | (0.059) | |
| $60,000 to 74,999 | -0.0051 | | -0.0054 | 0.075* | | | | 0.075* | 0.027 | | | 0.026 | | -0.023 | | -0.023 | 0.022 | | 0.022 | |
|  | (0.034) | | (0.034) | (0.035) | | | | (0.035) | (0.039) | | | (0.039) | | (0.038) | | (0.038) | (0.039) | | (0.039) | |
| $75,000 to 99,000 | 0.070** | | 0.069** | 0.022 | | | | 0.022 | 0.018 | | | 0.018 | | -0.037 | | -0.037 | 0.020 | | 0.018 | |
|  | (0.027) | | (0.027) | (0.031) | | | | (0.031) | (0.043) | | | (0.043) | | (0.038) | | (0.039) | (0.041) | | (0.040) | |
| $100,000 to 149,999 | 0.038^ | | 0.038^ | -0.011 | | | | -0.011 | 0.10** | | | 0.10** | | 0.065^ | | 0.065^ | 0.062^ | | 0.062^ | |
|  | (0.027) | | (0.027) | (0.035) | | | | (0.035) | (0.035) | | | (0.036) | | (0.042) | | (0.042) | (0.039) | | (0.039) | |
| $150,000 to 199,999 | 0.012 | | 0.012 | -0.013 | | | | -0.013 | 0.030 | | | 0.030 | | 0.073 | | 0.073 | 0.0021 | | 0.0019 | |
|  | (0.046) | | (0.046) | (0.039) | | | | (0.039) | (0.061) | | | (0.061) | | (0.059) | | (0.059) | (0.036) | | (0.036) | |
| $200,000 to 249,999 | 0.070* | | 0.071* | -0.0050 | | | | -0.0046 | 0.14*** | | | 0.14*** | | 0.026 | | 0.025 | 0.12** | | 0.13** | |
|  | (0.032) | | (0.033) | (0.048) | | | | (0.048) | (0.041) | | | (0.042) | | (0.042) | | (0.042) | (0.047) | | (0.047) | |
| $250,000 or more | -0.012 | | -0.012 | -0.039 | | | | -0.039 | 0.11* | | | 0.11* | | 0.011 | | 0.011 | 0.12** | | 0.12** | |
|  | (0.037) | | (0.037) | (0.054) | | | | (0.054) | (0.054) | | | (0.053) | | (0.050) | | (0.050) | (0.048) | | (0.047) | |
| Age | -0.024** | | -0.024** | -0.022* | | | | -0.022* | -0.014 | | | -0.014 | | -0.0030 | | -0.0031 | 0.00027 | | 0.0013 | |
|  | (0.0082) | | (0.0083) | (0.012) | | | | (0.012) | (0.011) | | | (0.011) | | (0.014) | | (0.014) | (0.012) | | (0.013) | |
| Same-sex * | | -0.024 | |  | | -0.0090 | | |  | | -0.023 | | |  | 0.011 | | |  | | -0.080* |
| Female |  | | (0.024) |  | | | | (0.029) |  | | | (0.037) | |  | | (0.037) |  | | (0.037) | |
| Ideology |  | |  | 0.058*** | | | | 0.058*** | 0.16*** | | | 0.16*** | | -0.044*** | | -0.044*** | 0.099*** | | 0.098*** | |
| (Con->Lib) |  | | | | (0.0092) | | (0.0092) | | | (0.013) | | | (0.013) | (0.012) | (0.012) | | | (0.011) | | (0.011) |
| Intercept | 0.49*** | | 0.49*** | 0.35** | | | | 0.35** | 0.21** | | | 0.21** | | 0.56*** | | 0.56*** | 0.51*** | | 0.49** | |
|  | (0.053) | | (0.051) | (0.15) | | | | (0.15) | (0.075) | | | (0.075) | | (0.084) | | (0.084) | (0.16) | | (0.17) | |
| *N* | 110127 | | 110127 | 109525 | | | | 109525 | 109230 | | | 109230 | | 109104 | | 109104 | 109157 | | 109157 | |
| R-squared | 0.061 | | 0.062 | 0.13 | | | | 0.13 | 0.21 | | | 0.21 | | 0.042 | | 0.042 | 0.12 | | 0.12 | |
| F | 4.49 | | 7.83 | 11.2 | | | | 15.0 | 20.2 | | | 19.5 | | 1.92 | | 2.11 | 7.99 | | 9.08 | |
| df_m | 28 | | 29 | 29 | | | | 30 | 29 | | | 30 | | 29 | | 30 | 29 | | 30 | |
| df_r | 110103 | | 110103 | 109501 | | | | 109501 | 109206 | | | 109206 | | 109080 | | 109080 | 109133 | | 109133 | |

*Note:* Standard errors in parentheses; ^ p<0.10; * p<0.05; ** p<0.01; *** p<0.001 (one-tailed)

**Table S3.2: Regression results continued, Same-Sex Female Couples**

|  | (11) | (12) | (13) | (14) | (15) | (16) | (17) | (18) | (19) | (20) |
| --- | --- | --- | --- | --- | --- | --- | --- | --- | --- | --- |
|  | Coll Ban Racist / Sexist Speech | Coll Ban Racist / Sexist Speech | Women’s Equal Pay | Women’s Equal Pay | US Not Intervene in Conf. | US Not Intervene in Conf. | Same-Sex Marriage | Same-Sex Marriage | Affirm. Act. in Coll. Admis. | Affirm. Act. in Coll. Admins. |
| Same-Sex Couple | -0.017 | -0.0043 | -0.0022 | 0.016 | 0.014 | 0.018 | 0.015 | -0.0054 | 0.016 | 0.053* |
|  | (0.02) | (0.03) | (0.01) | (0.02) | (0.01) | (0.02) | (0.02) | (0.03) | (0.02) | (0.03) |
| Female | 0.043* | 0.054*** | 0.062*** | 0.077*** | 0.024^ | 0.028*** | 0.086*** | 0.069*** | -0.042** | -0.011^ |
|  | (0.02) | (0.01) | (0.01) | (0.01) | (0.02) | (0.01) | (0.02) | (0.01) | (0.02) | (0.01) |
| Asian | -0.018 | -0.02 | 0.14* | 0.14* | 0.097 | 0.097 | 0.08 | 0.082 | -0.018 | -0.023 |
|  | (0.06) | (0.06) | (0.08) | (0.08) | (0.11) | (0.11) | (0.09) | (0.10) | (0.09) | (0.09) |
| Black | -0.0027 | -0.0051 | 0.12^ | 0.11^ | 0.19* | 0.19* | -0.055 | -0.051 | -0.057 | -0.064 |
|  | (0.05) | (0.05) | (0.08) | (0.08) | (0.11) | (0.11) | (0.10) | (0.10) | (0.09) | (0.09) |
| Hispanic | 0.05 | 0.047 | 0.16* | 0.16* | 0.18* | 0.18* | 0.085 | 0.089 | -0.083 | -0.091 |
|  | (0.05) | (0.06) | (0.08) | (0.08) | (0.11) | (0.11) | (0.10) | (0.10) | (0.09) | (0.10) |
| White | 0.0065 | 0.0044 | 0.16* | 0.16* | 0.14^ | 0.14^ | 0.069 | 0.072 | -0.12^ | -0.13^ |
|  | (0.05) | (0.05) | (0.08) | (0.08) | (0.11) | (0.11) | (0.10) | (0.10) | (0.09) | (0.09) |
| Other | -0.026 | -0.028 | 0.14* | 0.13* | 0.073 | 0.073 | 0.082 | 0.085 | 0.015 | 0.01 |
|  | (0.10) | (0.10) | (0.08) | (0.08) | (0.12) | (0.12) | (0.12) | (0.12) | (0.14) | (0.14) |
| Multi-racial | -0.024 | -0.027 | 0.14* | 0.14* | 0.15^ | 0.15^ | 0.084 | 0.088 | -0.048 | -0.056 |
|  | (0.05) | (0.05) | (0.08) | (0.08) | (0.11) | (0.11) | (0.10) | (0.10) | (0.09) | (0.09) |
| Gay | 0.20*** | 0.19*** | 0.076** | 0.075** | 0.066* | 0.065* | 0.18*** | 0.18*** | 0.026 | 0.024 |
|  | (0.05) | (0.05) | (0.03) | (0.03) | (0.04) | (0.04) | (0.03) | (0.03) | (0.06) | (0.06) |
| Lesbian | 0.033 | 0.033 | -0.11^ | -0.11^ | 0.0098 | 0.0095 | 0.059 | 0.06 | 0.080** | 0.078** |
|  | (0.05) | (0.05) | (0.07) | (0.07) | (0.06) | (0.06) | (0.05) | (0.05) | (0.03) | (0.03) |
| Bisexual | 0.014 | 0.014 | 0.00042 | 0.00039 | 0.012 | 0.012 | 0.089*** | 0.089*** | 0.063* | 0.063* |
|  | (0.04) | (0.04) | (0.02) | (0.02) | (0.02) | (0.02) | (0.02) | (0.02) | (0.03) | (0.03) |
| Queer | 0.075 | 0.074 | -0.13 | -0.13 | -0.028 | -0.028 | -0.06 | -0.057 | 0.12 | 0.11 |
|  | (0.12) | (0.12) | (0.12) | (0.12) | (0.10) | (0.10) | (0.10) | (0.10) | (0.11) | (0.11) |
| Other | -0.061 | -0.061 | 0.030* | 0.030* | -0.039 | -0.039 | 0.082* | 0.082* | 0.067* | 0.067* |
|  | (0.08) | (0.08) | (0.02) | (0.02) | (0.05) | (0.05) | (0.05) | (0.05) | (0.03) | (0.03) |
| First Gen | -0.033 | -0.033 | -0.016 | -0.016 | -0.035^ | -0.035^ | -0.0097 | -0.0094 | 0.036* | 0.035* |
|  | (0.03) | (0.03) | (0.02) | (0.02) | (0.02) | (0.02) | (0.02) | (0.02) | (0.02) | (0.02) |
| $10,000 to 14,999 | -0.061^ | -0.062^ | -0.066* | -0.067* | -0.071* | -0.071* | -0.013 | -0.011 | 0.022 | 0.019 |
|  | (0.04) | (0.04) | (0.03) | (0.03) | (0.03) | (0.03) | (0.04) | (0.04) | (0.04) | (0.04) |
| $15,000 to 19,999 | -0.014 | -0.015 | -0.051* | -0.051* | 0.0079 | 0.0078 | -0.014 | -0.013 | 0.055^ | 0.054^ |
|  | (0.05) | (0.05) | (0.03) | (0.03) | (0.03) | (0.03) | (0.04) | (0.04) | (0.04) | (0.04) |
| $20,000 to 24,999 | 0.0085 | 0.0084 | -0.041* | -0.041* | -0.013 | -0.014 | 0.046^ | 0.046^ | 0.013 | 0.013 |
|  | (0.05) | (0.05) | (0.02) | (0.02) | (0.03) | (0.03) | (0.03) | (0.03) | (0.04) | (0.04) |
| $25,000 to 29,999 | 0.10* | 0.10* | 0.013 | 0.013 | -0.064^ | -0.064^ | 0.027 | 0.027 | -0.016 | -0.017 |
|  | (0.05) | (0.05) | (0.02) | (0.02) | (0.04) | (0.04) | (0.04) | (0.04) | (0.05) | (0.05) |
| $30,000 to 39,999 | 0.0059 | 0.0057 | -0.03 | -0.03 | -0.062* | -0.062* | 0.032 | 0.032 | 0.028 | 0.028 |
|  | (0.05) | (0.05) | (0.03) | (0.03) | (0.03) | (0.03) | (0.03) | (0.03) | (0.03) | (0.03) |
| $40,000 to 49,999 | 0.045 | 0.045 | -0.033 | -0.033 | -0.014 | -0.014 | 0.057* | 0.057* | 0.036 | 0.035 |
|  | (0.04) | (0.04) | (0.03) | (0.03) | (0.04) | (0.04) | (0.03) | (0.03) | (0.04) | (0.04) |
| $50,000 to 59,999 | -0.032 | -0.032 | -0.02 | -0.02 | -0.088* | -0.088* | 0.024 | 0.024 | 0.013 | 0.014 |
|  | (0.06) | (0.06) | (0.02) | (0.02) | (0.05) | (0.05) | (0.03) | (0.03) | (0.04) | (0.04) |
| $60,000 to 74,999 | -0.013 | -0.013 | 0.015 | 0.015 | -0.040^ | -0.040^ | -0.0067 | -0.0064 | -0.061^ | -0.061^ |
|  | (0.04) | (0.04) | (0.02) | (0.02) | (0.03) | (0.03) | (0.06) | (0.06) | (0.05) | (0.05) |
| $75,000 to 99,000 | -7.1E-05 | -0.00056 | -0.015 | -0.016 | -0.078** | -0.078** | 0.038 | 0.039 | -0.048 | -0.049 |
|  | (0.04) | (0.04) | (0.02) | (0.02) | (0.03) | (0.03) | (0.03) | (0.03) | (0.04) | (0.04) |
| $100,000 to 149,999 | 0.025 | 0.025 | -0.0019 | -0.0019 | -0.051* | -0.051* | 0.068* | 0.068* | -0.038 | -0.038 |
|  | (0.04) | (0.04) | (0.02) | (0.02) | (0.03) | (0.03) | (0.03) | (0.03) | (0.04) | (0.04) |
| $150,000 to 199,999 | 0.01 | 0.01 | 0.0025 | 0.0025 | -0.061^ | -0.061^ | 0.027 | 0.028 | -0.052^ | -0.052^ |
|  | (0.06) | (0.06) | (0.02) | (0.02) | (0.05) | (0.05) | (0.05) | (0.05) | (0.04) | (0.04) |
| $200,000 to 249,999 | -0.12^ | -0.11^ | -0.0039 | -0.0026 | -0.099* | -0.099* | 0.085** | 0.084** | -0.015 | -0.012 |
|  | (0.08) | (0.08) | (0.02) | (0.02) | (0.05) | (0.05) | (0.03) | (0.03) | (0.06) | (0.06) |
| $250,000 or more | 0.015 | 0.015 | -0.032 | -0.032 | -0.16*** | -0.16*** | 0.085** | 0.085** | -0.12** | -0.12** |
|  | (0.05) | (0.05) | (0.03) | (0.03) | (0.04) | (0.04) | (0.04) | (0.04) | (0.05) | (0.05) |
| Age | 0.0019 | 0.0022 | 0.0024 | 0.0027 | -0.019* | -0.019* | -0.039** | -0.039** | 0.021* | 0.022* |
|  | (0.02) | (0.02) | (0.01) | (0.01) | (0.01) | (0.01) | (0.01) | (0.01) | (0.01) | (0.01) |
| Same-sex * |  | -0.021 |  | -0.029 |  | -0.0077 |  | 0.033 |  | -0.060* |
| Female |  | (0.04) |  | (0.02) |  | (0.03) |  | (0.03) |  | (0.03) |
| Ideology | 0.031** | 0.031** | 0.037*** | 0.036*** | 0.018^ | 0.018^ | 0.13*** | 0.13*** | 0.063*** | 0.062*** |
| (Con->Lib) | (0.01) | (0.01) | (0.01) | (0.01) | (0.01) | (0.01) | (0.02) | (0.02) | (0.01) | (0.01) |
| Intercept | 0.54*** | 0.53*** | 0.63*** | 0.62*** | 0.56*** | 0.56*** | 0.37*** | 0.37*** | 0.37*** | 0.35*** |
|  | (0.09) | (0.09) | (0.09) | (0.09) | (0.12) | (0.12) | (0.11) | (0.12) | (0.10) | (0.10) |
| *N* | 108706 | 108706 | 108975 | 108975 | 108754 | 108754 | 108667 | 108667 | 108539 | 108539 |
| R-squared | 0.03 | 0.03 | 0.082 | 0.083 | 0.05 | 0.05 | 0.23 | 0.23 | 0.1 | 0.1 |
| F | 2.28 | 4.19 | 6.88 | 14.4 | 3.67 | 5.02 | 30.9 | 33.5 | 6.47 | 6.58 |
| df_m | 29 | 30 | 29 | 30 | 29 | 30 | 29 | 30 | 29 | 30 |
| df_r | 108682 | 108682 | 108951 | 108951 | 108730 | 108730 | 108643 | 108643 | 108515 | 108515 |

*Note:* Standard errors in parentheses; ^ p<0.10; * p<0.05; ** p<0.01; *** p<0.001 (one-tailed)

**Table S3.3: Regression Results, Same-Sex Male Couples**

|  | (1) | (2) | (3) | (4) | (5) | (6) | (7) | (8) | (9) | (10) |
| --- | --- | --- | --- | --- | --- | --- | --- | --- | --- | --- |
|  | Ideology | Ideology | Racism in US | Racism in US | Abortion | Abortion | Coll. Ban Ext. Speech | Coll. Ban Ext. Speech | Marijuana | Marijuana |
| Same-Sex Couple | 0.012 | 0.0061 | -0.030 | -0.062* | -0.0058 | -0.025 | 0.035^ | 0.041^ | 0.036^ | 0.021 |
|  | (0.020) | (0.023) | (0.029) | (0.029) | (0.038) | (0.037) | (0.026) | (0.029) | (0.027) | (0.031) |
| Female | 0.057** | 0.044*** | 0.13** | 0.063*** | 0.043 | 0.0014 | -0.039^ | -0.026** | -0.052* | -0.086*** |
|  | (0.022) | (0.0056) | (0.045) | (0.012) | (0.058) | (0.013) | (0.028) | (0.0097) | (0.029) | (0.0093) |
| Asian | 0.41*** | 0.41*** | -0.0085 | -0.011 | 0.14** | 0.14** | 0.032 | 0.033 | -0.10* | -0.10* |
|  | (0.13) | (0.13) | (0.078) | (0.078) | (0.060) | (0.061) | (0.074) | (0.074) | (0.058) | (0.059) |
| Black | 0.41** | 0.41** | 0.26*** | 0.26*** | 0.063 | 0.064 | 0.028 | 0.027 | 0.064 | 0.065 |
|  | (0.14) | (0.14) | (0.077) | (0.078) | (0.074) | (0.074) | (0.080) | (0.080) | (0.077) | (0.078) |
| Hispanic | 0.43*** | 0.43*** | 0.040 | 0.039 | 0.063 | 0.063 | -0.092 | -0.092 | 0.023 | 0.023 |
|  | (0.13) | (0.13) | (0.092) | (0.092) | (0.087) | (0.087) | (0.082) | (0.081) | (0.062) | (0.063) |
| White | 0.36** | 0.36** | 0.060 | 0.053 | 0.19*** | 0.18*** | 0.0021 | 0.0037 | 0.038 | 0.035 |
|  | (0.13) | (0.13) | (0.073) | (0.073) | (0.055) | (0.055) | (0.070) | (0.070) | (0.053) | (0.054) |
| Other | 0.37** | 0.37** | 0.059 | 0.037 | 0.27** | 0.25** | 0.089 | 0.094 | -0.076 | -0.087 |
|  | (0.13) | (0.13) | (0.095) | (0.093) | (0.11) | (0.11) | (0.11) | (0.11) | (0.074) | (0.075) |
| Two or more race/ethnicity | 0.42*** | 0.42*** | 0.076 | 0.070 | 0.13* | 0.13* | 0.024 | 0.025 | 0.099* | 0.095* |
|  | (0.13) | (0.13) | (0.079) | (0.080) | (0.065) | (0.066) | (0.075) | (0.074) | (0.056) | (0.056) |
| Gay | 0.21*** | 0.20*** | -0.0087 | -0.013 | 0.028 | 0.026 | -0.041 | -0.040 | 0.057* | 0.055* |
|  | (0.018) | (0.017) | (0.079) | (0.076) | (0.056) | (0.055) | (0.034) | (0.033) | (0.031) | (0.031) |
| Lesbian | 0.13*** | 0.14*** | -0.018 | 0.034^ | 0.080^ | 0.11*** | 0.029 | 0.018 | 0.13*** | 0.16*** |
|  | (0.023) | (0.015) | (0.043) | (0.025) | (0.053) | (0.029) | (0.031) | (0.024) | (0.034) | (0.026) |
| Bisexual | 0.13*** | 0.13*** | -0.054 | -0.047 | 0.11* | 0.12* | -0.062^ | -0.063^ | 0.12** | 0.12** |
|  | (0.020) | (0.020) | (0.067) | (0.066) | (0.056) | (0.055) | (0.046) | (0.045) | (0.050) | (0.049) |
| Queer | 0.041 | 0.045 | -0.24^ | -0.22^ | -0.14 | -0.13 | 0.26** | 0.25** | 0.26*** | 0.27*** |
|  | (0.045) | (0.046) | (0.15) | (0.15) | (0.14) | (0.14) | (0.099) | (0.10) | (0.054) | (0.055) |
| Other | 0.12*** | 0.12*** | 0.11* | 0.11* | -0.037 | -0.040 | 0.0093 | 0.010 | -0.057 | -0.059 |
|  | (0.038) | (0.038) | (0.058) | (0.060) | (0.12) | (0.11) | (0.066) | (0.067) | (0.052) | (0.052) |
| First Gen | -0.025 | -0.024 | -0.021 | -0.014 | -0.031 | -0.027 | -0.012 | -0.013 | 0.042 | 0.046 |
|  | (0.020) | (0.021) | (0.042) | (0.041) | (0.053) | (0.053) | (0.045) | (0.045) | (0.042) | (0.042) |
| $10,000 to 14,999 | -0.11 | -0.11^ | 0.093 | 0.071 | -0.18* | -0.19* | 0.067 | 0.072 | -0.095^ | -0.11^ |
|  | (0.085) | (0.086) | (0.10) | (0.090) | (0.095) | (0.10) | (0.097) | (0.099) | (0.072) | (0.075) |
| $15,000 to 19,999 | -0.016 | -0.016 | 0.027 | 0.026 | 0.053 | 0.052 | -0.15^ | -0.15^ | 0.017 | 0.017 |
|  | (0.047) | (0.046) | (0.057) | (0.055) | (0.068) | (0.067) | (0.095) | (0.096) | (0.054) | (0.054) |
| $20,000 to 24,999 | 0.0020 | 0.0047 | -0.18* | -0.16* | -0.38** | -0.37** | -0.40** | -0.40** | -0.31* | -0.31* |
|  | (0.039) | (0.041) | (0.095) | (0.091) | (0.14) | (0.14) | (0.13) | (0.13) | (0.14) | (0.14) |
| $25,000 to 29,999 | 0.033 | 0.032 | 0.089 | 0.085 | -0.17^ | -0.18^ | -0.094^ | -0.093^ | 0.033 | 0.031 |
|  | (0.053) | (0.053) | (0.075) | (0.074) | (0.12) | (0.12) | (0.062) | (0.063) | (0.073) | (0.072) |
| $30,000 to 39,999 | -0.060 | -0.066 | -0.099 | -0.13^ | -0.038 | -0.058 | -0.15** | -0.14** | -0.029 | -0.045 |
|  | (0.099) | (0.099) | (0.079) | (0.088) | (0.11) | (0.12) | (0.052) | (0.057) | (0.11) | (0.11) |
| $40,000 to 49,999 | -0.024 | -0.022 | -0.13* | -0.12^ | -0.12* | -0.11* | -0.074 | -0.077 | 0.065 | 0.071 |
|  | (0.043) | (0.044) | (0.080) | (0.080) | (0.068) | (0.067) | (0.060) | (0.060) | (0.095) | (0.096) |
| $50,000 to 59,999 | 0.010 | 0.011 | 0.030 | 0.036 | 0.029 | 0.032 | -0.017 | -0.018 | 0.043 | 0.046 |
|  | (0.047) | (0.047) | (0.057) | (0.057) | (0.069) | (0.068) | (0.049) | (0.049) | (0.056) | (0.056) |
| $60,000 to 74,999 | 0.049 | 0.051 | 0.052 | 0.064 | -0.16* | -0.15* | -0.13* | -0.14* | -0.14* | -0.14* |
|  | (0.043) | (0.044) | (0.061) | (0.060) | (0.089) | (0.088) | (0.080) | (0.080) | (0.080) | (0.081) |
| $75,000 to 99,000 | 0.0034 | 0.0046 | -0.0058 | 0.00028 | -0.081 | -0.077 | -0.13* | -0.13* | 0.092^ | 0.095^ |
|  | (0.046) | (0.046) | (0.061) | (0.061) | (0.073) | (0.070) | (0.064) | (0.064) | (0.062) | (0.062) |
| $100,000 to 149,999 | -0.0074 | -0.0080 | 0.017 | 0.014 | 0.038 | 0.036 | -0.14** | -0.14** | 0.035 | 0.033 |
|  | (0.043) | (0.043) | (0.066) | (0.063) | (0.067) | (0.063) | (0.048) | (0.049) | (0.057) | (0.056) |
| $150,000 to 199,999 | -0.066 | -0.064 | 0.053 | 0.059 | 0.089^ | 0.093^ | -0.086^ | -0.087* | 0.11^ | 0.11^ |
|  | (0.057) | (0.057) | (0.065) | (0.064) | (0.065) | (0.063) | (0.053) | (0.053) | (0.070) | (0.071) |
| $200,000 to 249,999 | 0.018 | 0.020 | 0.0038 | 0.012 | -0.018 | -0.013 | -0.060 | -0.062 | -0.0042 | 0.00010 |
|  | (0.046) | (0.046) | (0.075) | (0.073) | (0.080) | (0.078) | (0.061) | (0.061) | (0.060) | (0.061) |
| $250,000 or more | -0.047 | -0.047 | 0.0022 | 0.0046 | -0.033 | -0.031 | -0.12* | -0.12* | 0.083^ | 0.085^ |
|  | (0.052) | (0.052) | (0.077) | (0.078) | (0.077) | (0.077) | (0.056) | (0.056) | (0.063) | (0.063) |
| Age | -0.037** | -0.038** | -0.0064 | -0.0075 | -0.015 | -0.016 | 0.014 | 0.014 | -0.016 | -0.016 |
|  | (0.013) | (0.013) | (0.021) | (0.021) | (0.016) | (0.016) | (0.013) | (0.013) | (0.016) | (0.016) |
| Same-sex * |  | 0.029 |  | 0.15* |  | 0.094 |  | -0.031 |  | 0.076^ |
| Female |  | (0.051) |  | (0.088) |  | (0.12) |  | (0.060) |  | (0.059) |
| Ideology |  |  | 0.075*** | 0.074*** | 0.18*** | 0.18*** | -0.050*** | -0.050*** | 0.094*** | 0.094*** |
| (Con->Lib) |  |  | (0.014) | (0.014) | (0.019) | (0.019) | (0.016) | (0.016) | (0.015) | (0.015) |
| Intercept | 0.27* | 0.27* | 0.42*** | 0.44*** | -0.045 | -0.032 | 0.66*** | 0.66*** | 0.25** | 0.27** |
|  | (0.15) | (0.15) | (0.13) | (0.13) | (0.11) | (0.11) | (0.11) | (0.11) | (0.097) | (0.098) |
| *N* | 109783 | 109783 | 109179 | 109179 | 108884 | 108884 | 108761 | 108761 | 108812 | 108812 |
| R-squared | 0.14 | 0.14 | 0.20 | 0.21 | 0.24 | 0.25 | 0.10 | 0.10 | 0.18 | 0.19 |
| F | 19.6 | 29.0 | 9.08 | 12.7 | 15.6 | 16.3 | 3.73 | 4.22 | 12.2 | 14.5 |
| df_m | 28 | 29 | 29 | 30 | 29 | 30 | 29 | 30 | 29 | 30 |
| df_r | 109759 | 109759 | 109155 | 109155 | 108860 | 108860 | 108737 | 108737 | 108788 | 108788 |

*Note:* Standard errors in parentheses; ^ p<0.10; * p<0.05; ** p<0.01; *** p<0.001 (one-tailed)

**Tables S3.4: Regression Results, Same-Sex Male Couples (cont.)**

|  | (11) | (12) | (13) | (14) | (15) | (16) | (17) | (18) | (19) | (20) |
| --- | --- | --- | --- | --- | --- | --- | --- | --- | --- | --- |
|  | Coll Ban Racist / Sexist Speech | Coll Ban Racist / Sexist Speech | Women’s Equal Pay | Women’s Equal Pay | US Not Intervene in Conf. | US Not Intervene in Conf. | Same-Sex Marriage | Same-Sex Marriage | Affirm. Act. in Coll. Admis. | Affirm. Act. in Coll. Admins. |
| Same-Sex Couple | -0.044^ | -0.055* | -0.017 | -0.025 | -0.026 | -0.018 | 0.00039 | 0.0017 | 0.020 | -0.0017 |
|  | (0.027) | (0.031) | (0.021) | (0.026) | (0.026) | (0.024) | (0.029) | (0.030) | (0.026) | (0.029) |
| Female | 0.089** | 0.065*** | 0.100*** | 0.082*** | 0.011 | 0.029** | 0.063* | 0.066*** | 0.046* | -0.00038 |
|  | (0.033) | (0.011) | (0.016) | (0.011) | (0.040) | (0.0096) | (0.032) | (0.011) | (0.028) | (0.0090) |
| Asian | 0.083 | 0.082 | 0.013 | 0.012 | -0.074^ | -0.073 | 0.17** | 0.17** | 0.041 | 0.039 |
|  | (0.070) | (0.071) | (0.039) | (0.038) | (0.057) | (0.058) | (0.061) | (0.061) | (0.063) | (0.063) |
| Black | 0.16* | 0.16* | 0.055 | 0.056 | 0.054 | 0.053 | 0.15* | 0.15* | 0.0020 | 0.0030 |
|  | (0.092) | (0.092) | (0.044) | (0.044) | (0.072) | (0.073) | (0.080) | (0.080) | (0.079) | (0.079) |
| Hispanic | 0.11^ | 0.11^ | 0.034 | 0.033 | -0.12* | -0.12* | 0.19** | 0.19** | 0.094^ | 0.093^ |
|  | (0.078) | (0.079) | (0.054) | (0.053) | (0.060) | (0.061) | (0.070) | (0.069) | (0.069) | (0.069) |
| White | 0.069 | 0.066 | 0.010 | 0.0083 | -0.075^ | -0.073^ | 0.17** | 0.17** | 0.016 | 0.011 |
|  | (0.067) | (0.068) | (0.038) | (0.037) | (0.052) | (0.052) | (0.058) | (0.058) | (0.059) | (0.059) |
| Other | 0.17* | 0.16* | 0.027 | 0.022 | 0.026 | 0.032 | 0.21** | 0.21** | 0.072 | 0.057 |
|  | (0.093) | (0.093) | (0.059) | (0.057) | (0.084) | (0.087) | (0.078) | (0.078) | (0.074) | (0.074) |
| Two or more race/ethnicity | 0.13* | 0.13* | 0.013 | 0.011 | -0.029 | -0.027 | 0.18** | 0.18** | 0.0054 | 0.0012 |
|  | (0.071) | (0.071) | (0.044) | (0.043) | (0.054) | (0.055) | (0.061) | (0.061) | (0.064) | (0.064) |
| Gay | 0.095** | 0.093** | 0.067*** | 0.066*** | 0.031 | 0.032 | 0.13*** | 0.13*** | -0.017 | -0.020 |
|  | (0.032) | (0.032) | (0.020) | (0.020) | (0.026) | (0.026) | (0.027) | (0.027) | (0.050) | (0.052) |
| Lesbian | 0.0095 | 0.028 | -0.056* | -0.043* | 0.023 | 0.0094 | 0.13*** | 0.12*** | 0.010 | 0.047* |
|  | (0.037) | (0.027) | (0.028) | (0.024) | (0.035) | (0.024) | (0.031) | (0.021) | (0.030) | (0.024) |
| Bisexual | -0.041 | -0.039 | -0.036 | -0.034 | 0.0018 | 0.00015 | 0.12*** | 0.12*** | 0.062^ | 0.066^ |
|  | (0.042) | (0.041) | (0.040) | (0.039) | (0.046) | (0.046) | (0.025) | (0.025) | (0.045) | (0.046) |
| Queer | 0.27*** | 0.27*** | -0.37** | -0.36** | -0.29** | -0.30** | -0.20^ | -0.20^ | -0.18* | -0.17* |
|  | (0.083) | (0.084) | (0.15) | (0.15) | (0.12) | (0.12) | (0.15) | (0.15) | (0.080) | (0.082) |
| Other | -0.0025 | -0.0040 | 0.0071 | 0.0060 | 0.053 | 0.055 | 0.11** | 0.11** | 0.086^ | 0.083^ |
|  | (0.071) | (0.071) | (0.030) | (0.030) | (0.048) | (0.048) | (0.042) | (0.042) | (0.057) | (0.058) |
| First Gen | 0.040 | 0.043 | -0.0072 | -0.0053 | -0.019 | -0.021 | -0.029 | -0.029 | -0.034 | -0.030 |
|  | (0.036) | (0.037) | (0.026) | (0.026) | (0.028) | (0.028) | (0.051) | (0.051) | (0.039) | (0.039) |
| $10,000 to 14,999 | 0.075 | 0.067 | 0.030 | 0.024 | 0.041 | 0.047 | -0.21* | -0.21* | 0.0015 | -0.014 |
|  | (0.086) | (0.082) | (0.064) | (0.061) | (0.12) | (0.12) | (0.12) | (0.12) | (0.11) | (0.098) |
| $15,000 to 19,999 | -0.015 | -0.015 | 0.034 | 0.034 | -0.038 | -0.038 | -0.071 | -0.071 | -0.20** | -0.21** |
|  | (0.066) | (0.065) | (0.058) | (0.058) | (0.051) | (0.051) | (0.080) | (0.080) | (0.071) | (0.070) |
| $20,000 to 24,999 | -0.19** | -0.18** | -0.093^ | -0.089^ | -0.42** | -0.43** | -0.21** | -0.21** | -0.27*** | -0.26*** |
|  | (0.077) | (0.077) | (0.066) | (0.066) | (0.15) | (0.16) | (0.086) | (0.087) | (0.084) | (0.083) |
| $25,000 to 29,999 | -0.15* | -0.15* | 0.021 | 0.020 | -0.065 | -0.064 | 0.028 | 0.028 | -0.13* | -0.13* |
|  | (0.075) | (0.074) | (0.079) | (0.079) | (0.060) | (0.060) | (0.066) | (0.066) | (0.059) | (0.060) |
| $30,000 to 39,999 | -0.14^ | -0.15* | 0.024 | 0.016 | -0.049 | -0.040 | -0.11 | -0.11 | -0.24* | -0.26** |
|  | (0.085) | (0.089) | (0.056) | (0.055) | (0.058) | (0.066) | (0.13) | (0.13) | (0.10) | (0.11) |
| $40,000 to 49,999 | -0.11^ | -0.11^ | -0.074 | -0.071 | -0.094^ | -0.097^ | -0.041 | -0.041 | -0.085 | -0.076 |
|  | (0.072) | (0.072) | (0.059) | (0.059) | (0.062) | (0.062) | (0.054) | (0.055) | (0.068) | (0.070) |
| $50,000 to 59,999 | 0.024 | 0.026 | 0.052 | 0.053 | -0.084* | -0.086* | 0.014 | 0.013 | -0.21** | -0.20** |
|  | (0.070) | (0.071) | (0.058) | (0.059) | (0.049) | (0.049) | (0.053) | (0.053) | (0.067) | (0.068) |
| $60,000 to 74,999 | 0.037 | 0.041 | 0.0037 | 0.0069 | -0.13* | -0.13* | -0.080 | -0.080 | -0.27*** | -0.26*** |
|  | (0.080) | (0.080) | (0.063) | (0.063) | (0.061) | (0.062) | (0.087) | (0.087) | (0.081) | (0.082) |
| $75,000 to 99,000 | -0.096^ | -0.094 | -0.048 | -0.047 | -0.059 | -0.060 | -0.068 | -0.068 | -0.30*** | -0.30*** |
|  | (0.074) | (0.074) | (0.068) | (0.068) | (0.057) | (0.057) | (0.070) | (0.070) | (0.071) | (0.071) |
| $100,000 to 149,999 | -0.058 | -0.059 | 0.019 | 0.019 | -0.15** | -0.15** | 0.081^ | 0.081^ | -0.21*** | -0.21*** |
|  | (0.063) | (0.063) | (0.061) | (0.061) | (0.057) | (0.056) | (0.053) | (0.053) | (0.063) | (0.063) |
| $150,000 to 199,999 | -0.023 | -0.020 | 0.0021 | 0.0038 | -0.058 | -0.060 | 0.11^ | 0.11^ | -0.28*** | -0.28*** |
|  | (0.077) | (0.078) | (0.077) | (0.076) | (0.062) | (0.063) | (0.068) | (0.068) | (0.073) | (0.073) |
| $200,000 to 249,999 | -0.061 | -0.058 | 0.068 | 0.070 | -0.16** | -0.16** | 0.100* | 0.099* | -0.21** | -0.20** |
|  | (0.078) | (0.078) | (0.059) | (0.059) | (0.062) | (0.062) | (0.058) | (0.058) | (0.074) | (0.076) |
| $250,000 or more | -0.060 | -0.059 | 0.062 | 0.062 | -0.16** | -0.16** | 0.13** | 0.13** | -0.20** | -0.19** |
|  | (0.076) | (0.077) | (0.062) | (0.062) | (0.053) | (0.053) | (0.057) | (0.057) | (0.065) | (0.066) |
| Age | 0.0017 | 0.0014 | -0.024* | -0.025* | -0.012 | -0.011 | -0.018^ | -0.018^ | 0.020 | 0.019 |
|  | (0.014) | (0.014) | (0.013) | (0.013) | (0.012) | (0.012) | (0.014) | (0.014) | (0.015) | (0.016) |
| Same-sex * |  | 0.053 |  | 0.039 |  | -0.040 |  | -0.0064 |  | 0.11* |
| Female |  | (0.073) |  | (0.035) |  | (0.080) |  | (0.071) |  | (0.055) |
| Ideology | -0.0041 | -0.0044 | 0.054*** | 0.054*** | 0.070*** | 0.070*** | 0.15*** | 0.15*** | 0.084*** | 0.083*** |
| (Con->Lib) | (0.018) | (0.018) | (0.017) | (0.017) | (0.014) | (0.014) | (0.017) | (0.017) | (0.018) | (0.018) |
| Intercept | 0.59*** | 0.59*** | 0.76*** | 0.77*** | 0.62*** | 0.61*** | 0.11 | 0.11 | 0.35** | 0.36** |
|  | (0.12) | (0.12) | (0.10) | (0.10) | (0.094) | (0.095) | (0.094) | (0.094) | (0.12) | (0.12) |
| *N* | 108361 | 108361 | 108634 | 108634 | 108412 | 108412 | 108326 | 108326 | 108196 | 108196 |
| R-squared | 0.078 | 0.079 | 0.22 | 0.22 | 0.16 | 0.17 | 0.30 | 0.30 | 0.19 | 0.19 |
| F | 3.25 | 6.77 | 10.1 | 16.5 | 6.25 | 8.58 | 43.6 | 49.2 | 6.98 | 8.15 |
| df_m | 29 | 30 | 29 | 30 | 29 | 30 | 29 | 30 | 29 | 30 |
| df_r | 108337 | 108337 | 108610 | 108610 | 108388 | 108388 | 108302 | 108302 | 108172 | 108172 |

*Note:* Standard errors in parentheses; ^ p<0.10; * p<0.05; ** p<0.01; *** p<0.001 (one-tailed)
