# Supplementary material for: Potential differences between the political attitudes of people with same-sex parents and people with different-sex parents: An exploratory assessment of first-year college students
Source: PLoS One. 2021 Feb 25;16(2):e0246929. doi: 10.1371/journal.pone.0246929 (PMC7906383; doi:10.1371/journal.pone.0246929)
Supplement: S4 Appendix — (DOCX) [file pone.0246929.s004.docx]

**Supporting Information 4: Replication of results excluding Black respondents for children of same-sex female couples**

There was a high number of Black respondents reporting to have same-sex female parents. HERI administrators also noticed a higher propensity for people with SS female parents to racially self-identify as Black. They proposed this may be due to Black respondents reporting the sex of their mother and grandmother or aunt residing in the same household. Though, the patterns are consistent with demographic research on same-sex couple households (Gates 2013), we replicated our analyses excluding Black respondents from people SS female parents, and the results are consistent. We re-estimated the propensity score model excluding black respondents (children of same-sex female couples ​*N* ​= 444; children of different-sex couples ​*N* ​= 124,312). The estimation fit statistics similarly suggested that the model was not an incorrect specification (Hansen’s-​*J*​ = 9.26 * 10^−8^ ). Figure S4.1 plots the mean absolute differences between children of same-sex female couples and children of different-sex couples before and after weighting. The weighting procedure minimizes observable differences between these two groups. As in the main text, the propensity score weights are multiplied by the sampling weights.

Table S4.1 shows the demographic differences between people with same-sex parents and people with different-sex parents both before and after weighting on the propensity score. Respondents who have same-sex female parents are less heterosexual, more female, lower income, less likely racially identify as white, and more likely to racially identify as a racial or ethnic group that’s not Latino or Asian compared to their counterparts from different-sex couples. Differences between the two groups are minimized by the weighting, and there are no significant differences.

Table S4.2 shows the means of the dependent variables by whether or not people report SS female parents or DS parents, prior to and after matching. With the weighted and unmatched data, people who have SS parents are more progressive on numerous measures: political ideology, beliefs in race discrimination being a problem in the United States, same-sex marriage, and affirmative action in college admissions. These differences are substantially reduced after matching. Similar to the main text, the differences remain with people with SS female parents are still more likely to believe that race discrimination is a problem in the United States and more favorable of affirmative action in college admissions compared to people with DS parents.

Table S4.3 reports similar results by gender. The results replicate the findings of the main text: when differences do exist between people who have SS female parents and DS parents, the effect of SS parents tends to be more progressive policy opinions. Males who have SS female parents are more progressive in their attitudes, and females who have SS female parents are more only more likely to view race discrimination as a problem in the United States. These results are consistent with the results reported in the main text.

**Figure S4.1: Balance tests before and after weighting**


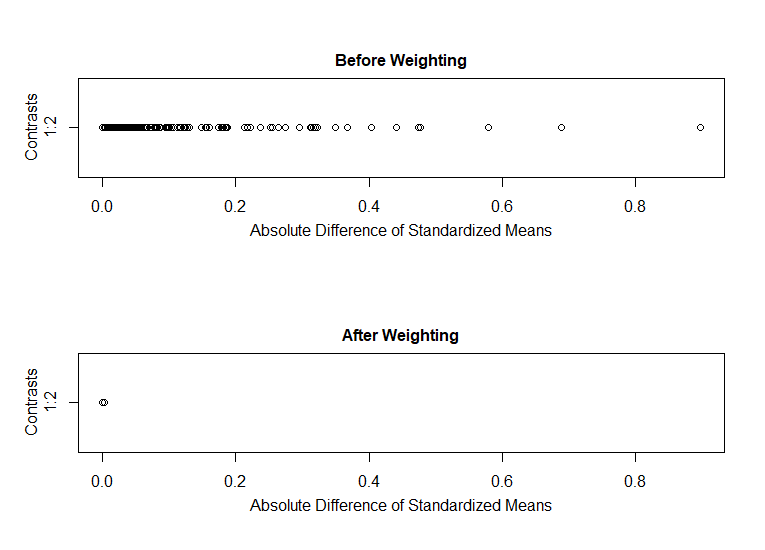


**Table S4.1: Summary statistics**

|  | Same-sex female couples | |  |
| --- | --- | --- | --- |
|  | Probability Weights | | Final Weights |
| Variable | Same-sex | Different-sex | Different-sex |
| Heterosexual | 0.79 (0.02) | 0.85 (0.001)* | 0.79 (0.005) |
| Female | 0.62 (0.02) | 0.54 (0.002)* | 0.62 (0.005) |
| First generation | 0.15 (0.02) | 0.15 (0.001) | 0.15 (0.005) |
| Income | 7.71 (0.23) | 9.59 (0.012)* | 7.71 (0.04) |
| Age | 3.40 (0.05) | 3.31 (0.002) | 3.40 (0.02) |
| White | 0.47 (0.03) | 0.62 (0.002)* | 0.47 (0.005) |
| Black | -- | -- | -- |
| Latino | 0.12 (0.02) | 0.10 (0.001) | 0.12 (0.003) |
| Asian | 0.15 (0.02) | 0.11 (0.001) | 0.15 (0.003) |
| Other | 0.26 (0.03) | 0.15 (0.001)* | 0.26 (0.005) |

*Note*​: Same-sex female couples ​*N =*​ 444; comparison sample ​*N* ​= 124,312; means are reported; standard errors are in the parentheses; significance tests are for differences between children of same-sex couples and children of different-sex couples; *p<0.05 (two-tailed).

**Table S4.2: Political views**

|  | Same-sex female couples | | | |
| --- | --- | --- | --- | --- |
|  | Unmatched (weighted) | | Matched | *N* |
|  | Same- sex | Different-sex | Different-sex |  |
| Ideology  (Con->Lib) | .59  (.01) | .53  (.0008)* | .58  (.002) | 116,706 |
| Racial discrimination a problem | .80  (.02) | .72  (.001)* | .75  (.002)* | 118,855 |
| Abortion should be legal | .64  (.02) | .60  (.001)* | .65  (.003) | 118,146 |
| Colleges ban extreme speech | .43  (.02) | .45  (.001) | .44  (.003) | 117,756 |
| Marijuana Legalization | .56  (.02) | .52  (.001) | .56  (.003) | 117,707 |
| College prohibit  racist/sexist speech | .67  (.02) | .67  (.001) | .68  (.003) | 116,857 |
| Gender workplace equality | .94  (.01) | .92  (.0007)* | .93  (.002) | 117,345 |
| US should intervene in conflicts | .65  (.02) | .63  (.001) | .66  (.003) | 116,580 |
| Same-sex marriage | .86  (.02) | .79  (.001)* | .84  (.003) | 116,896 |
| Affirmative action in college admissions | .57  (.02) | .48  (.001)* | .53  (.003)* | 116,242 |

Note: Significance tests are for differences between children of same-sex couples and children of different-sex couples; standard errors are in the parentheses; *p<0.05 (one-tailed).

Table S4.3: Political views among comparing people with SS female parents

|  | Females | | | Males | | | |
| --- | --- | --- | --- | --- | --- | --- | --- |
|  | Unmatched (weighted) | | Matched | Unmatched (weighted) | | Matched |  |
|  | Same- sex | Different- sex | Different- sex | Same- sex | Different- sex | Different- sex | *N* |
| Ideology  (Conservative to Liberal) | .58  (.02) | .55  (.001) | .60  (.002) | .60  (.02) | .51 (.001)* | .54  (.003)* | 116,706 |
| Racial discrimination is a problem in the US | .82  (.02) | .75  (.001)* | .78  (.003)* | .76  (.03) | .69  (.001)* | .71  (.003)* | 118,855 |
| Abortion should be legal | .62  (0.03) | .60  (.002) | .66  (.005) | .69  (.03) | .59  (.002)* | .64  (.004) | 118,146 |
| Colleges should ban extreme speech | .46  (.03) | .44  (.001) | .43  (.004) | .40  (.03) | .46  (.002)* | .45  (.005) | 117,756 |
| Marijuana legalization | .49  (.03) | .49  (.002) | .53  (.004) | .68  (.03) | .56  (.002)* | .59  (.005)* | 117,707 |
| College should prohibit  racist/sexist speech | .70  (.03) | .70  (.002) | .71  (.004) | .61  (.03) | .63  (.002) | .64  (.004) | 116,857 |
| Gender workplace equality | .95  (.01) | .95  (.001) | .96  (.002) | .90  (.02) | .87  (.001) | .88  (.002) | 117,345 |
| US should not intervene in conflicts | .67 (.02) | .65  (.001) | .67  (.004) | .61  (.03) | .61  (.001) | .63  (.003) | 116,580 |
| Same-sex marriage | .87  (.03) | .83  (.002) | .87  (.004) | .85  (.02) | .74  (.002)* | .79  (.004)* | 116,896 |
| Affirmative action in college admissions | .55  (.03) | .49  (.002)* | .54  (.003) | .59 (.03) | .48  (.002)* | .51  (.004)* | 116,242 |

Note: All variables scaled from zero to one with higher values indicating more progressive responses. Differences between people with same-sex parents and different-sex parents; standard errors are in the parentheses; *p<0.05 (one-tailed).
