# Supplementary material for: Potential differences between the political attitudes of people with same-sex parents and people with different-sex parents: An exploratory assessment of first-year college students
Source: PLoS One. 2021 Feb 25;16(2):e0246929. doi: 10.1371/journal.pone.0246929 (PMC7906383; doi:10.1371/journal.pone.0246929)
Supplement: S5 Appendix — (DOCX) [file pone.0246929.s005.docx]

**S5 Appendix. Partisanship in the General Social Survey**

The cumulative General Social Survey 1972-2018, contains survey data of adults in the United States, including sexual orientation, parentage, and partisanship. Reported in Table S6.1 is the partisan breakdown by parentage and sexual orientation. The results show that LGB parents are more likely to be political independents while LGB non-parents are more likely to be strong Democrats. Thus, there are political differences among LGB people depending on whether they are parents. These analyses were performed in the publicly available Statistical Data Archive hosted by the University of California at Berkeley (<https://sda.berkeley.edu/sdaweb/analysis/?dataset=gss18>). The row variable was “PARTYID,” the column variables was “YNKIDST,” and the control variable was “LGB_DUMMY.”

Table S6.1. Partisanship by Sexual Orientation and Parentage.

|  | LGB | | Non-LGB | |
| --- | --- | --- | --- | --- |
|  | Non-parent | Parent | Non-parent | Parent |
| Strong Democrat | 22.6% | 25.2% | 14.0% | 16.3% |
| Weak Democrat | 21.5% | 11.7% | 18.0% | 15.8% |
| Lean Democrat | 19.9% | 17.5% | 16.8% | 11.9% |
| Independent | 16.4% | 24.6% | 17.7% | 17.4% |
| Lean Republican | 5.2% | 5.9% | 10.6% | 10.3% |
| Weak Republican | 5.2% | 7.1% | 11.9% | 14.5% |
| Strong Republican | 2.9% | 5.6% | 7.6% | 11.5% |
| Other | 6.4% | 2.3% | 3.4% | 2.3% |
| *N* | 294 | 185 | 2,757 | 7,370 |
| *F* (*df*, *df*) | 2.10 (7, 1078)^a^ | | 10.94 (7, 1078)^b^ | |

Note: ^a^*p* = 0.04; ^b^*p* < .001.
