# Supplementary material for: Potential differences between the political attitudes of people with same-sex parents and people with different-sex parents: An exploratory assessment of first-year college students
Source: PLoS One. 2021 Feb 25;16(2):e0246929. doi: 10.1371/journal.pone.0246929 (PMC7906383; doi:10.1371/journal.pone.0246929)
Supplement: S6 Appendix — (DOCX) [file pone.0246929.s006.docx]

**S6 Appendix: Unweighted Results**

Results were reported relying on probability and final weights incorporating the complex survey design of the Freshman Survey. The weights adjust point estimates to generalize to the target population, and other design features are incorporated to accurately estimate standard errors. In this appendix, unweighted summary data by people with SS and DS parents are provided in Table S7.1, political views are in Table S7.2, and by gender in Tables S7.3-S7.4. We note that any differences reported in these results assume simple random sampling, which may underestimate standard errors. We provide these unweighted estimates as a point of comparison.

The results are largely consistent with what is reported based on the probability weights. People SS parents are less heterosexual and slightly older than people with DS parents. People with SS male parents are far more male, and people with SS female parents have lower household incomes and are more racially and ethnically diverse than both people with SS male couples and DS couples. The political attitudes continue to show that the sample is overall more politically progressive. People with SS female parents are more progressive in their racial attitudes than people with DS parents, and they tend to be politically more progressive overall. People with SS male parents remain more conservative on women’s equal pay and stand out in their favorability of legalizing marijuana use. By gender, females with SSF parents are more politically progressive on numerous outcomes, while females with SSM parents only differ in their views of race discrimination in the US and legalizing marijuana use. For males, we continue to observe that males with SS male parents are more conservative on women’s equal pay while males with SSF parents are more politically progressive on a variety of measures.

**Table S7.1. Unweighted Demographics by Household Type.**

|  | **Household Type** | | |
| --- | --- | --- | --- |
|  | **SS Female Couples** | **SS Male Couples** | **DS Couples** |
| **Variables** | **Mean (*SE*)** | **Mean (*SE*)** | **Mean (*SE*)** |
| **Heterosexual** | 0.78 (0.02) | 0.73 (0.03) | 0.86 (0.001)^b,c^ |
| **Female** | 0.62 (0.02) | 0.21 (0.03)^a^ | 0.56 (0.001)^b,c^ |
| **First generation** | 0.19 (0.02) | 0.12 (0.02)^a^ | 0.14 (0.001)^b^ |
| **Income** | 6.93 (0.17) | 9.97 (0.28)^a^ | 9.61 (0.01)^b^ |
| **Age Group** | 3.41 (0.03) | 3.46 (0.07) | 3.30 (0.002)^b,c^ |
| **White** | 0.36 (0.02) | 0.51 (0.04)^a^ | 0.59 (0.001)^b,c^ |
| **Black** | 0.27 (0.02) | 0.07 (0.02)^a^ | 0.08 (0.001)^b^ |
| **Latino** | 0.08 (0.01) | 0.05 (0.02)^a^ | 0.08 (0.001)^c^ |
| **Asian** | 0.13 (0.01) | 0.20 (0.03)^a^ | 0.13 (0.001)^c^ |
| **Other** | 0.16 (0.02) | 0.17 (0.03) | 0.12 (0.001)^b^ |
| *N* | 602 | 176 | 134,142 |

SS = same-sex; DS = different-sex.

^a^Difference between people with SS female parents and people with SS male parents is significant at *p* < .05 (one-tailed).

^b^Difference between people with SS female parents and people with DS parents is significant at *p* < .05 (one-tailed).

^c^Difference between people with SS male parents and people with DS parents is significant at *p* < .05 (one-tailed).

**Table S7.2: Unweighted political views**

|  | | Household Type | | |
| --- | --- | --- | --- | --- |
|  | **SS Female Couples** | **SS Male Couples** | **DS Couples** |  |
| **Variables** | **Mean (*SE*)** | **Mean (*SE*)** | **Mean (*SE*)** |  |
| Ideology  (Conservative to Liberal) | | .60 (.01) | .57 (.02)^a^ | .54 (.001)^b^ |
| Racial discrimination is a problem in the US | | .82 (.01) | .72 (.02)^a^ | .74 (.001)^b^ |
| Abortion should be legal | | .62 (0.02) | .63 (.03) | .60 (.001) |
| Colleges should ban extreme speech | | .41 (.01) | .48 (.03)^a^ | .45 (.001)^b^ |
| Marijuana legalization | | .58 (.01) | .60 (.03) | .53 (.001)^b,c^ |
| College should prohibit  racist/sexist speech | | .67 (.01) | .60 (.03)^a^ | .68 (.001)^c^ |
| Gender workplace equality | | .92 (.009) | .86 (.02)^a^ | .92 (.001)^c^ |
| US should not intervene in conflicts | | .67 (.01) | .60 (.02)^a^ | .64 (.001)^b^ |
| Same-sex marriage | | .83 (.01) | .79 (.02) | .79 (.001)^b^ |
| Affirmative action in college admissions | | .59 (.01) | .53 (.02)^a^ | .50 (.001)^b^ |

Note: All variables scaled from zero to one with higher values indicating more progressive responses.

SS = same-sex; DS = different-sex.

^a^Difference between people with SS female parents and people with SS male parents is significant at *p* < .05 (one-tailed).

^b^Difference between people with SS female parents and people with DS parents is significant at *p* < .05 (one-tailed).

^c^Difference between people with SS male parents and people with DS parents is significant at *p* < .05 (one-tailed).

**Table S7.3: Unweighted political views, females**

|  | | Household Type | | |
| --- | --- | --- | --- | --- |
|  | **SS Female Couples** | **SS Male Couples** | **DS Couples** |  |
| **Variables** | **Mean (*SE*)** | **Mean (*SE*)** | **Mean (*SE*)** |  |
| Ideology  (Conservative to Liberal) | | .61 (.01) | .65 (.03) | .57 (.001)^b,c^ |
| Racial discrimination is a problem in the US | | .84 (.01) | .81 (.05) | .77 (.001)^b^ |
| Abortion should be legal | | .61 (0.02) | .70 (.07) | .61 (.001) |
| Colleges should ban extreme speech | | .41 (.02) | .45 (.05) | .44 (.001)^b^ |
| Marijuana legalization | | .53 (.02) | .63 (.06) | .50 (.001)^b,c^ |
| College should prohibit  racist/sexist speech | | .68 (.02) | .66 (.06) | .71 (.001) |
| Gender workplace equality | | .94 (.009) | .95 (.03) | .95 (.001)^b^ |
| US should not intervene in conflicts | | .69 (.01) | .65 (.05) | .66 (.001)^b^ |
| Same-sex marriage | | .87 (.01) | .87 (.05) | .83 (.001)^b^ |
| Affirmative action in college admissions | | .57 (.02) | .57 (.05) | .50 (.001)^b^ |

Note: All variables scaled from zero to one with higher values indicating more progressive responses.

SS = same-sex; DS = different-sex.

^a^Difference between people with SS female parents and people with SS male parents is significant at *p* < .05 (one-tailed).

^b^Difference between people with SS female parents and people with DS parents is significant at *p* < .05 (one-tailed).

^c^Difference between people with SS male parents and people with DS parents is significant at *p* < .05 (one-tailed).

**Table S7.4: Unweighted political views, males**

|  | | Household Type | | |
| --- | --- | --- | --- | --- |
|  | **SS Female Couples** | **SS Male Couples** | **DS Couples** |  |
| **Variables** | **Mean (*SE*)** | **Mean (*SE*)** | **Mean (*SE*)** |  |
| Ideology  (Conservative to Liberal) | | .59 (.02) | .55 (.02) | .52 (.001)^b^ |
| Racial discrimination is a problem in the US | | .78 (.02) | .70 (.03)^a^ | .71 (.001)^b^ |
| Abortion should be legal | | .64 (0.03) | .62 (.03) | .59 (.001)^b^ |
| Colleges should ban extreme speech | | .41 (.02) | .48 (.03)^a^ | .46 (.001)^b^ |
| Marijuana legalization | | .66 (.02) | .60 (.03) | .57 (.001)^b^ |
| College should prohibit  racist/sexist speech | | .65 (.02) | .58 (.03)^a^ | .64 (.001)^c^ |
| Gender workplace equality | | .88 (.02) | .83 (.03) | .87 (.001)^c^ |
| US should not intervene in conflicts | | .63 (.02) | .59 (.03) | .62 (.001) |
| Same-sex marriage | | .78 (.02) | .78 (.03) | .75 (.001) |
| Affirmative action in college admissions | | .63 (.02) | .52 (.03)^a^ | .49 (.001)^b^ |

Note: All variables scaled from zero to one with higher values indicating more progressive responses.

SS = same-sex; DS = different-sex.

^a^Difference between people with SS female parents and people with SS male parents is significant at *p* < .05 (one-tailed).

^b^Difference between people with SS female parents and people with DS parents is significant at *p* < .05 (one-tailed).

^c^Difference between people with SS male parents and people with DS parents is significant at *p* < .05 (one-tailed).

**References**

Gates, Gary J. 2013. ​*LGBT Parenting in the United States*​. Los Angeles, CA: The Williams Institute.
